# Novel Quinazoline Derivatives Inhibit Splicing of Fungal Group II Introns

**DOI:** 10.1021/acschembio.4c00631

**Published:** 2025-01-17

**Authors:** Olga Fedorova, Michelle Luo, G. Erik Jagdmann, Michael C. Van Zandt, Luke Sisto, Anna Marie Pyle

**Affiliations:** † Howard Hughes Medical Institute, Chevy Chase, Maryland 20815, United States; ‡ Department of Molecular, Cellular and Developmental Biology, Yale University, New Haven, Connecticut 06520, United States; § Department of Chemistry, Yale University, New Haven, Connecticut 06520, United States; ∥ New England Discovery Partners, Branford, Connecticut 06405, United States

## Abstract

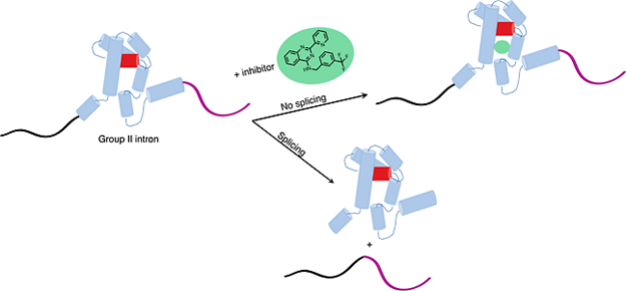

We report the discovery of small molecules that target the RNA tertiary structure of self-splicing group II introns and display potent antifungal activity against yeasts, including the major public health threat *Candida parapsilosis*. High-throughput screening efforts against a yeast group II intron resulted in an inhibitor class which was then synthetically optimized for enhanced inhibitory activity and antifungal efficacy. The most highly refined compounds in this series display strong, gene-specific antifungal activity against *C. parapsilosis*. This work demonstrates the utility of combining advanced RNA screening methodologies with medicinal chemistry pipelines to identify high-affinity ligands targeting RNA tertiary structures with important roles in human health and disease.

## Introduction

Many RNA molecules are involved in metabolic processes that affect human health. Large RNAs often fold into highly complex tertiary structures that can be specifically recognized by small molecules.^1,2^ These structurally unique RNA elements represent attractive targets for drug discovery.^3−8^ Selective RNA targeting is not without precedent: for example, it is well-known that many antibiotics target rRNA molecules in bacteria, thereby inhibiting the synthesis of bacterial proteins. The aminoglycosides (streptomycin, neomycin, kanamycin B, spectinomycin, and paromomycin) and tetracyclines (tetracycline and tigecycline) bind specific tertiary structures within 16S rRNA, while macrolides (erythromycin, azithromycin, spiramycin, and telithromycin) and oxazolidinones (linezolid and eperezolid) target well-defined pockets within 23S rRNA.^9^ Bacterial riboswitches, which typically bind naturally occurring metabolites, can also bind drug-like compounds with high affinity, rendering them promising targets for new antibiotic development.^10−12^ Viruses have also been targeted with small-molecule inhibitors as their genomes often contain elaborate functional RNA motifs (reviewed in^7^). Targeted viral elements include the TAR and RRE stem-loops within HIV RNA,^13−15^ the HCV IRES element,^16−18^ the SARS CoV-2 frameshifting pseudoknot,^19^ and the influenza A virus RNA promoter.^20,21^ Other studies have focused on host-specific RNAs such as regulatory microRNAs^22,23^ and disease-associated repeats.^24−26^ Despite these developments, there are few cases in which inhibitors have been obtained using methodical *de novo* targeting approaches that involve target elucidation, high-throughput screening (HTS), SAR, and optimization. Such approaches enable the targeting of any desired RNA structural motif, rather than the few RNA elements that present themselves serendipitously, and they are built from medicinal chemistry methodologies that have long powered the pharmaceutical industry.

Fungal organisms contain many RNA tertiary structures and RNA processing pathways that are not shared with humans, making them particularly promising targets for antifungal drug design. RNA targeting of fungi represents an important area of investigation because drug-resistant fungal infections are an increasingly significant threat to public health. Deadly systemic and invasive fungal infections are now prevalent in immunocompromised patients including those with chronic respiratory diseases, cancer, AIDS, and recipients of organ transplants. Available drugs are few as there are only four major classes of antifungal drugs in clinical use: polyenes, azoles, echinocandins, and allylamines, and most are highly toxic. Given these relatively limited options, the development of new antifungal treatments is crucial.

Like most fungal organisms, fungal pathogens within the highest priority disease groups (as defined by the World Health Organization) contain self-splicing group I and group II introns within mitochondrial rRNA and genes involved in respiration.^27,28^ Splicing of these introns is required for proper mitochondrial function in fungi and is therefore essential for the survival of pathogenic organisms. Given that self-splicing group I and II introns are absent in higher eukaryotes, they represent attractive targets for the development of new antifungal drugs. Previous studies have demonstrated that certain types of small molecules can inhibit self-splicing of some group I and group II introns.^29−34^ In earlier studies, we utilized an iterative medicinal chemistry approach to identify selective group II intron inhibitors that dramatically obstruct the growth of yeasts such as *Candida parapsilosis* by inhibiting splicing of a group II intron in cytochrome oxidase subunit genes.^33^ Inspired by this earlier success, here we employ an expanded library of 1,50,000 compounds to screen for additional intron-binding scaffolds. Combining screening with SAR and optimization, we identify new quinazoline derivatives that actively inhibit the splicing of a target group II intron in vitro and in vivo. Like compounds from our previous study, these compounds also dramatically and selectively inhibit the growth of yeasts such as the pathogen *C. parapsilosis*. In this way, we demonstrate that the same RNA tertiary structure can be efficiently targeted by multiple, structurally diverse small-molecule scaffolds, any one of which can potentially be developed into new therapeutic classes.

## Results and Discussion

### High-Throughput Screening Identification of the Novel Group II Intron Inhibitor Scaffold

**Figure 1:**
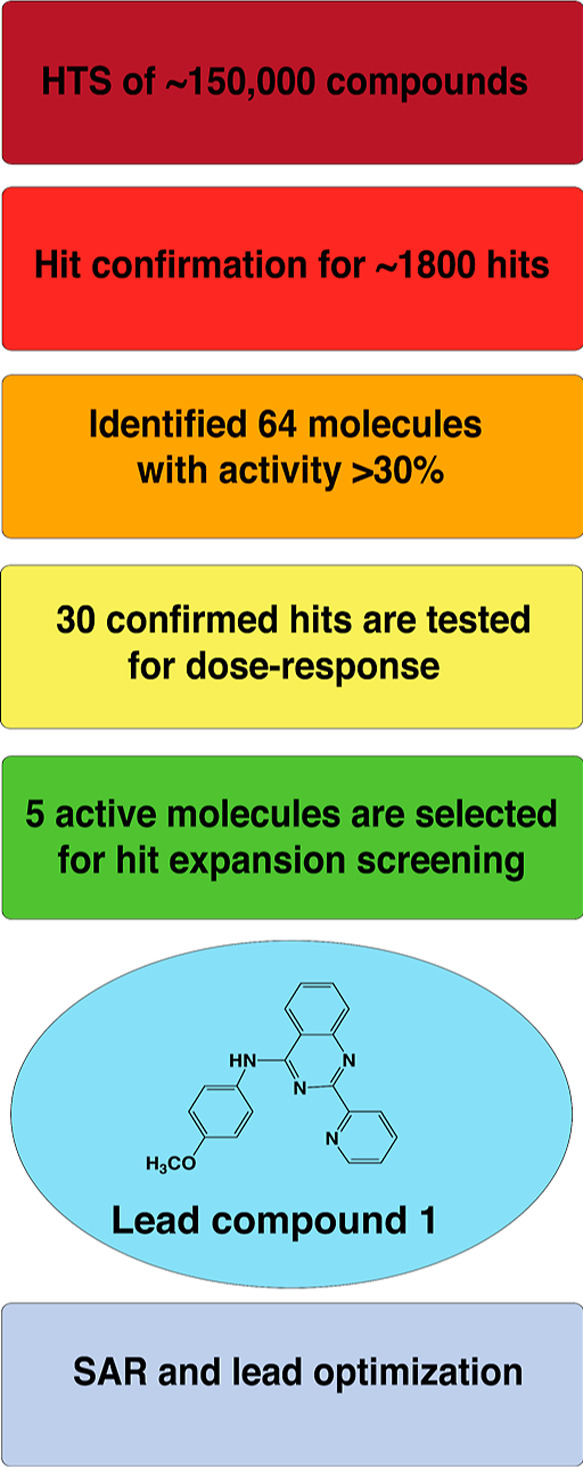
Experimental pipeline for discovery of new group II intron inhibitors.

To identify new inhibitors of the *C. parapsilosis* group II intron, we followed a classical pipeline beginning with chemical screening, followed by hit confirmation, dose–response testing of confirmed hits, hit expansion, SAR, and lead scaffold optimization (Figure 1). HTS was carried out by Charles River Laboratories (CRL) using their proprietary small-molecule library of 1,50,000 compounds and an intron activity assay adapted from previous studies (see Materials and Methods section).^33^ HTS identified 1817 preliminary hits, of which only 64 active molecules were confirmed to have inhibitory activity >30%. The five scaffolds with the highest activity in the dose–response assay (IC_50_ = 5–6 μM) were then used as the basis for in silico hit expansion. Selected scaffolds were screened against 6,25,000 compounds from the CRL diversity library to identify similar compounds outside the original screen, expanding the list of possible hits.^35^ Hit expansion revealed five different classes of compounds, of which the quinazolines proved most potent (IC_50_ = 3–5 μM). Quinazoline derivatives are often lead compounds in drug development given their antimicrobial, antitumor, and anti-inflammatory properties.^36^ Quinazolines were previously shown to bind RNA molecules such as the theophylline aptamer^37^ and influenza A virus RNA promoter.^20^ In addition, 4-aminoquinazolines are well-known kinase inhibitors.^38^ A representative of this family, compound **1** (Figure 1, Table S1), was one of the most promising hits from the screen (IC_50_ = 3 μM).

## SAR and Optimization

**Figure 2:**
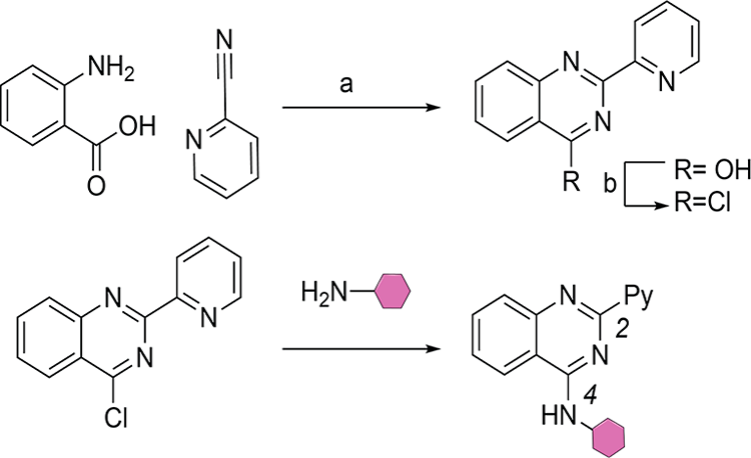
General synthesis of quinazoline analogues (a) NaOMe, MeOH, 65 °C, 16 h, 78%. (b) DMF, SOCl_2_, 75 °C, 2 h, 91%. Purple hexagon indicates aryl and benzylamines.

The 4-aminoquinazoline scaffold was selected, and analogues were synthesized via a short divergent route (Figure 2).

**Figure 3:**
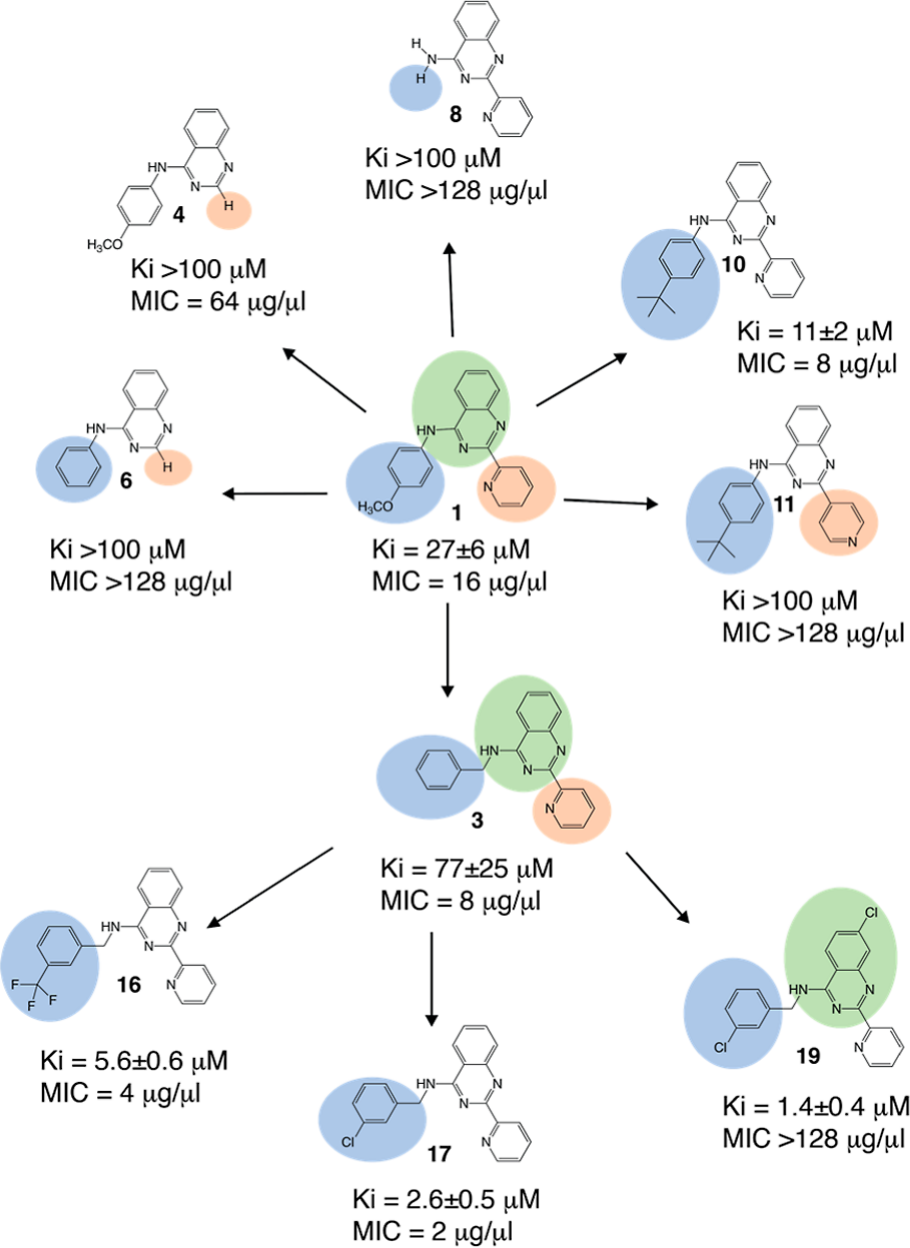
Summary of the SAR data. The effect of substituents on the *K*_i_ for the in vitro splicing of the ai5γ intron and minimum inhibitory concentration (MIC) for *C. parapsilosis.* Three functional regions of parental scaffolds **1** and **3** [4-aminoquinazoline, phenyl (or benzyl), and pyridine moieties] are highlighted in green, blue, and orange, respectively. Highlighted regions of the compound analogues are modified relative to the parental compounds.

The screening hit, compound **1**, had 2-pyridine and *para*-methoxyphenyl substituents at the 2- and 4-amino positions, respectively (Figure 1, Table S1). We next replaced the *para*-methoxyphenyl substituent of compound **1** with a *meta-*methoxybenzyl group to furnish compound **2**. This additional methylene group provided a greater reach and flexibility for the aromatic ring, while the shifted methoxy group remained closer to the original orientation of compound **1** (compound **2**, Figure 3 and Table S1).

**Figure 4:**
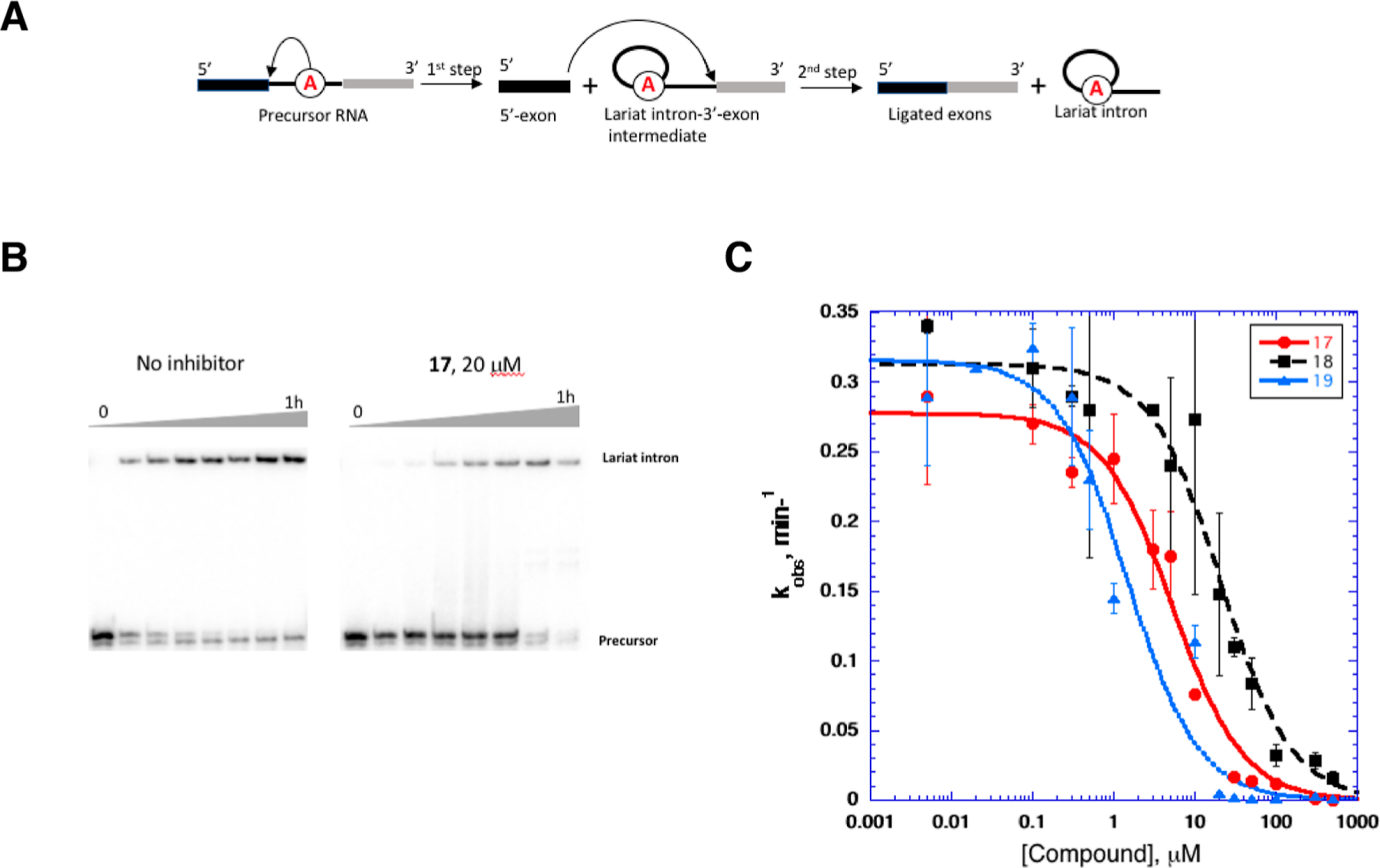
Inhibition of the ai5γ group II intron splicing by new inhibitors in vitro. (A) Schematic of the ai5γ intron splicing. (B) PAGE analysis of representative time courses of the ai5γ intron splicing in the absence (left) and in the presence (right) of compound **17**. (C) Representative *K*_i_ curves were obtained by plotting the rate constants (*k*_obs_ values) vs concentration of inhibitors. Data represent average of *n* = 2 independent experiments. Error bars are s.e.m. For comparison, the *K*_i_ values for the previously published active inhibitors intronistat A and intronistat B are 2.1 ± 0.2 and 0.36 ± 0.02 μM, respectively.^33^

Removal of the methoxy group from compound **2** provided similarly potent benzyl compound **3** (compare compounds **2** and **3**, Table S1), prompting us to find the minimal pharmacophore. We iteratively removed substituents from the 2- and 4-amino quinazoline positions (compounds **4**–**9**, Figure 3, Table S1) and studied the inhibitory activity of the resulting compounds with a radioanalytic splicing assay (see Materials and Methods section, Figure 4A). We observed that the removal of either ring resulted in a complete loss of inhibitory activity (Figure 3, Table S1), suggesting that the minimal pharmacophore requires both the pyridine and amino-aromatic groups (compare compounds **3** and **8**, Figure 3, Table S1). The required amino-aromatic group likely participates in a π–π stacking or π H-bonding interactions as these interactions are known to contribute substantially to overall binding between nucleic acids, small molecules, and larger biopolymers.

With a minimal pharmacophore established, further exploration of amino-aromatic groups began. The first compound possessing significant in vitro intron inhibition contained a *tert*-butylphenyl group (compound **10**, Table S1), hinting at the impact of large lipophilic groups on potency. Interestingly, repositioning of the pyridine nitrogen from an *ortho*- to a *para*-position completely abolishes activity (compare compounds **10** and **11**, Table S1), suggesting that the pyridine nitrogen in the *ortho*-position is critical for interaction with the ribozyme active site. Considering this, a favorable interaction likely requires two H-bond acceptors arranged in a bidentate fashion. Intron active sites are rich in metal ions, and the minimal pharmacophore is an analogue of the widely used 2,2′-bipyridine ligand. Inhibition of group II intron splicing by previously reported “intronistat” compounds^33^ involves coordinating metal ions in the intron active site by their pyrogallol moiety.^39^ Metal chelation by quinazoline and pyridine nitrogens is therefore one possible mechanism for intron inhibition by this new series of compounds.

Next, we found that the aniline moiety in compound **1** could be substituted with a more complex 1,3-benzodioxole-5 amino group (compound **12**), resulting in a moderate (3-fold) increase in inhibitory activity. Similarly, analogues based on compound **3**, containing constrained benzylamine derivatives 1,2,3,4-tetrahydroisoquinoline (compound **13**) and 1,3-dihydroisoindole (compound **14**), resulted in a moderate (3–4-fold) increase in inhibitory activity (Table S1). Some aspect of these fused rings, be it their rigidity or extended π systems, likely improved π-based interactions with the intron.

To further optimize the scaffold and identify better inhibitors, we evaluated various amino-aromatic substituents with a particular focus on the steric bulk. Building upon *tert*-butylphenyl compound **10**, *tert-*butyl and a trifluoromethylbenzyl groups in the same position improved inhibitory activity more than 10-fold (compounds **15** and **16**, Figure 3, Table S1) relative to unsubstituted benzyl compound **3**. Likewise, the *meta*-chlorobenzyl analogue proved most active, exhibiting a *K*_i_ value 30-fold lower than compound **3** (compound **17**, Figures 3 and 4, Table S1). Considering this observation and the previous trends related to π interactions, the bulky substituents may act as wedges, fixing the π system in a certain position or plane for optimal interactions.

We also explored direct halogenation of the quinazoline moiety. Interestingly, the effect of halogenation on potency was not consistent and depended on the benzylic substituents in the remainder of the molecule. A chloroquinazoline core caused a four-fold drop in the inhibitory activity when coupled with a trifluoromethylbenzyl group (compare compounds **16** and **18**, Table S1). However, a *meta*-chlorobenzyl substituent paired with the chloroquinazoline core resulted in the most active inhibitor (compound **19**, Figure 3, Table S1) with the *K*_i_ of 1.4 ± 0.4 μM. Notably, inhibitory activity of compounds **17** and **19** is comparable to that of previously published high affinity ligands with low micromolar *K*_i_ values.^33^

The initial HTS hit, compound **1**, possessed a moderate *K*_i_ (27 ± 6 μM), and subsequent SAR optimization revealed a clear trend between the electronics of the amino-aromatic substituent and intron binding. Electron rich and neutral amino-aromatic groups tended to have higher *K*_i_ measurements (compounds **1**–**3**), while the best binding was obtained with compounds bearing large, electron poor, benzyl groups (compounds **16** and **17**)*.* Other observed structural trends outweighed this electronic effect, such as fused and sterically hindered amino-aromatic groups (compounds **10, 12–15**). Chlorination of the quinazoline core altered this relationship, as well (compounds **18** and **19**).

**Figure 5:**
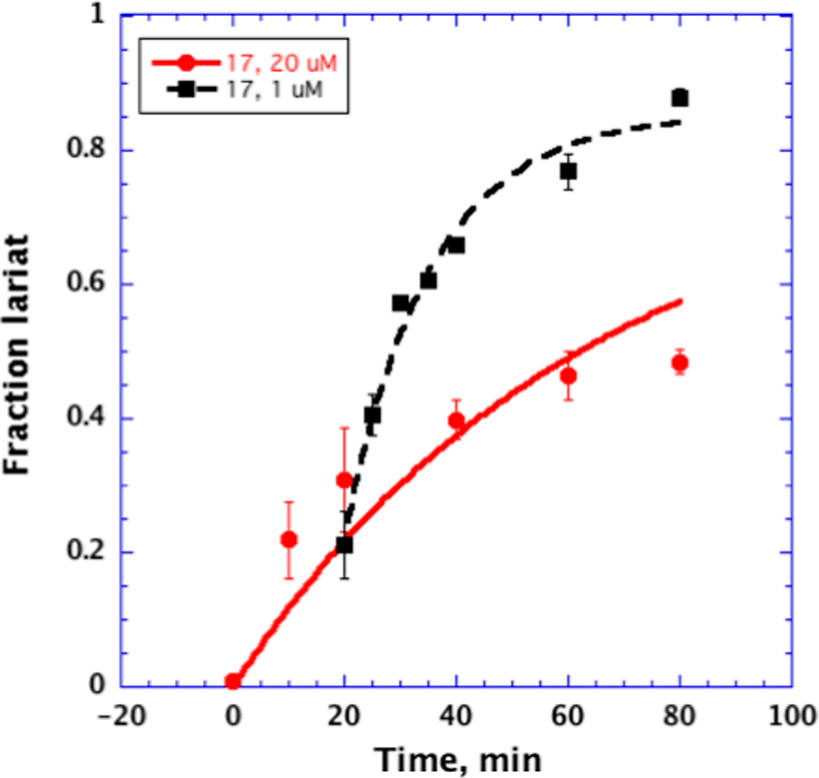
Inhibition of the ai5γ intron splicing by **17** at 20 μM (red) and after dilution from 20 μM to 1 μM (black). Data represent average of *n* = 3 independent experiments, error bars are s.e.m.

To determine whether the inhibitors bind reversibly to the intron, we conducted a variation of a previously described pulse-chase experiment.^33^ For this experiment, we used compound **17**, which is among the most active inhibitors (Figures 3 and 4, Table S1). After initiating reaction in the presence of the inhibitor, we observed that a 20-fold dilution of the reaction mixture (from 20 to 1 μM inhibitor) results in a corresponding increase in splicing rate constant (Figure 5), indicating that the compound inhibits splicing in a reversible manner. Taken together, these data suggest that the new family of compounds represent a promising class of group II intron inhibitors. Accordingly, we investigated whether they inhibited the target *in cellulo* and ultimately impeded fungal growth.

### Inhibition of Growth of *C. parapsilosis* by New Compounds

The COX1 gene of the fungal pathogen *C. parapsilosis* contains a single group IIB intron^40^ that is similar to that of the ai5γ group II intron from *Saccharomyces cerevisiae*.^33^ Previously identified ai5γ intron inhibitors are potent antifungal agents that interfere with the growth of *C. parapsilosis*, displaying MIC values of 2–4 μg/mL.^33^ Upon testing growth inhibition of *C. parapsilosis* with the quinazoline compounds, we observed that numerous active *S. cerevisiae* ai5γ intron splicing inhibitors (compounds **10**, **14**, **16**, and **17**) exhibited comparable MIC values in *C. parapsilosis*, ranging from 2 to 8 μg/mL (Figure 3, Table S1). Compound **17** was the most active inhibitor of *C. parapsilosis* growth (MIC = 2 μg/mL). However, a few of the less active compounds in the *S. cerevisiae* system (e.g., **5** and **7**, see Table S1) weakly inhibited the growth of *C. parapsilosis* with MIC values of 8–16 μg/mL, suggesting some differences in the active-site of the *C. parapsilosis* intron. This is not surprising given the differences among these two species of yeast.^41−43^ At the same time, certain compounds such as **3**, **10**, and **16** displayed a marked preference for inhibition of the *C. parapsilosis* intron.

That growth inhibition is specifically attributable to compound engagement with the selected group II intron target established by experiments using mutant yeast strains that lack the intron altogether (and therefore are not dependent on its splicing). Compounds **14** and **16**, which inhibit the growth *ofC. parapsilosis* (MIC values of 8 and 4 μg/mL, respectively), also demonstrate selective inhibition of the *S. cerevisiae* system, which enabled us to utilize genetic tools that have been designed for the study of *S. cerevisiae* For example, compound **14** inhibits the growth of the WT *S. cerevisiae* strain eight-fold more strongly than the corresponding COX1 intronless *S. cerevisiae* strain^44^ (MIC 4 μg/mL for the WT strain and 32 μg/mL for the intronless strain). In the case of compound **16**, MIC values for the *S. cerevisiae* WT and the intronless strain were 16 and 64 μg/mL, respectively, underscoring the specific role of the intron in mediating observed inhibitory effects.

### Directly Monitoring Inhibitor Effect on *C. parapsilosis* COX1 mRNA Splicing in Fungal Cells

**Figure 6:**
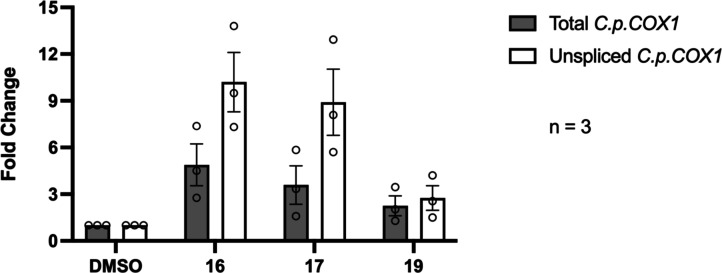
*C. parapsilosis* COX1 exhibits a splicing defect in the presence of targeted intron inhibitors **16** and **17**. *C. parapsilosis* was grown and treated with DMSO vehicle only, inactive compound (**19**), or active compound (**16**, **17**). Relative levels of total and unspliced *C. parapsilosis* COX1 transcripts are indicated by RT-qPCR quantification of amplicons covering the exon (total) or intron-exon junction from the group IIB intron (unspliced). Mean values and s.e.m. from *n* = 3 independent experiments are shown with *C. parapsilosis* PGK1 as a standard. For comparison, in the presence of intronistat B, the levels of unspliced *C. parapsilosis* COX1 transcripts are 1.5-fold higher than those of total *C. parapsilosis* COX1.^33^

To directly monitor the influence of inhibitors on the splicing of specific genes in the *C. parapsilosis* group IIB intron in fungal cells, we used qRT-PCR to monitor levels of splicing for the *C. parapsilosis* COX1 precursor mRNA, and unrelated genes, in the presence of active compounds **16** and **17** and inactive compound **19**. Compounds **16** and **17** induced moderate defects in the splicing of the COX1 gene (Figure 6). Levels of unspliced *C. parapsilosis* COX1 mRNA increased relative to levels of total *C. parapsilosis* COX1 mRNA in the presence of the active compounds but remained unchanged in the presence of the in vivo inactive compound **19** (Figure 6). Importantly, these effects were not seen in the splicing of genes that do not contain a group IIB intron, such as nuclear genes (data not shown). This is consistent with previous studies in which qRT-PCR enabled us to monitor similar *C. parapsilosis* COX1 group IIB intron splicing defects upon administration of targeted inhibitors, indicating a level of specificity comparable to that of previously characterized compounds.^33^

### Toxicity Studies in Human Cell Lines

To establish relative toxicity in human cells, we measured the effects of the most potent compounds in human HEK-293T cells by determining IC_50_ values for cell growth inhibition. Many of the quinazoline compounds exhibited various levels of cytotoxicity, with IC_50_ values for growth inhibition ranging from 1 to 10 μg/mL. This is perhaps not surprising because 4-aminoquinazoline is a privileged and biologically active scaffold that has been shown to be effective in applications that include chemotherapy.^45,46^ Multiple biological targets may be impacted as 4-aminoquinazolines are known to inhibit certain protein enzymes.^47^ Specifically, 2- pyridinyl analogues have been shown to inhibit serine kinases (ALK5) and phosphodiesterases.^47,48^ Of note to their implementation in this study, the most promising analogues are less cytotoxic than amphotericin B, which is the standard of care for antifungal infection in many clinical settings (comparative IC_50_ values in HEK-293T cells for compounds **16**, **17**, and amphotericin B are 5.6 ± 0.1, 3.9 ± 0.1, and 2.6 ± 0.2 μg/mL, respectively). Indeed, for amphotericin B, toxicity has typically been mitigated through the development of specific formulations, for example, lipid preparations,^49,50^ and the rational design of improved analogues that retain efficacy but lack toxicity,^51^ both of which are approaches that could be implemented in this case as well. While our goal is the discovery of systemically tolerated treatments for invasive fungal infections, compounds such as quinazolines remain applicable for topical administration or development in agricultural settings.

### Outlook and Perspectives

By targeting a specific class of group II introns within genes essential for yeast respiration, we have obtained a potent new class of antifungal agents through a classic screening and medicinal chemistry development pipeline, exemplifying the growing field of *de novo* RNA drug targeting.^52,53^ Unlike many serendipitously discovered RNA binders or natural product ligands, the quinazoline compounds reported here and in earlier studies are synthetically tractable, drug-like molecules that can be further optimized to improve pharmacological properties and enable manufacture. Perhaps the most significant aspect of the work is that we present a second series of compounds targeted to group II introns, thereby demonstrating that functional RNA target engagement can be achieved through a diversity of molecular interaction strategies and compound classes. The work therefore exemplifies the promise of RNA targeting in the development of antimicrobials and therapies for the modulation of the transcriptome.

## Materials and Methods

### Yeast Strains

*C. parapsilosis* [strain American Type Culture Collection (ATCC) 22019] was purchased from ATCC and cultured according to the manufacturer’s instructions. *S. cerevisiae* wild-type (NP40-36a) and mtDNA intronless (XPM46) strains were a gift from Dr. Thomas Fox (Dept. of Molecular Biology and Genetics, Cornell University).^44^

### RNA Preparation

#### In Vitro Transcription

Internally ^32^P-labeled ai5γ intron RNA with short exons^54^ was in vitro transcribed in the presence of ^32^P-α-UTP using T7 RNA polymerase and purified on a 5% denaturing polyacrylamide gel as described.^55^ Large-scale transcription of the D135 ribozyme, utilized for HTS, was carried out as previously described.^56^

#### Oligonucleotide Synthesis and Labeling

The RNA oligonucleotide substrate for HTS, containing the last 17 nucleotides of the native 5′-exon and the first two nucleotides of the ai5γ intron,^33^ was synthesized on a MerMade 12 RNA-DNA synthesizer (BioAutomation) using TBDMS RNA phosphoramidites (TxBio), aminomodifier C6dT, and Black Hole Quencher 2 CPG (Glen Research). For base deprotection, the oligonucleotide was treated with 30% ammonium hydroxide (JT Baker) for 24 h at RT. The 2’–OH deprotection and oligonucleotide purification were carried out as described.^57,58^ Fluorescent labeling of the substrate with the NHS ester of AlexaFluor 555 was carried out as described.^33^

#### HTS, Hit Confirmation, and Hit Expansion

HTS was carried out by CRL using the fluorescently labeled substrate, unlabeled D135 ribozyme (see above), and a proprietary library of ∼1,50,000 compounds using assay conditions previously described,^33^ with the following adaptations. The D135 ribozyme (20 nM final concentration) was first prefolded in a buffer of 50 mM MOPS, pH 7.5, 50 mM MgCl_2_, and 500 mM KCl at 37 °C for 30 min. Library compounds were then added at 12.5 μM final concentration followed by addition of the substrate (to 15 nM final concentration), and the reaction mixtures were incubated at 37 °C for 40 min. Reactions were was quenched by addition of urea (2.67 M final) and analyzed as described. Compounds exhibiting >25.71% inhibition [average (Av) + 3*standard deviation (StDev)] were considered significant hits suitable for subsequent hit confirmation (1817 compounds total). To that end, new plates containing only hit compounds were created and screened twice. Confirmed hits with an inhibitory activity >30% (64 compounds total) were then carried forward for dose–response analysis by CRL, using the same assay as that used for HTS. The dose–response analysis was carried out using 10 compound concentrations. The highest concentration was 100 μM, from which a 1:3 serial dilution was performed. Dose–response experiments were performed in duplicate to ensure reproducibility. The same compounds were also subjected to LCMS purity analysis. Most of the selected compounds except for two passed the purity test with >75% purity. Hit expansion with related molecules was conducted on the five most active scaffolds (IC_50_ = 5–6 μM) with the purpose of finding additional hits. The resulting hits (30 compounds) were tested for dose–response by CRL as above, five most active compounds were resynthesized by New England discovery partners and tested for activity. The most active compound 1 was then subjected to SAR studies.

### Determination of Splicing Inhibition Constants

Splicing inhibition constants were determined as previously described^33,34^ with the following change in the experimental procedure: reactions were carried out in a buffer of 50 mM MOPS pH 7.5, 100 mM MgCl_2_, and 500 mM KCl at 42 °C. Aliquots of the reaction mixture were withdrawn at different time points, quenched, and analyzed on a 5% denaturing polyacrylamide gel as previously described.^55^ Inhibition constants were determined as previously described.^33,34^ Experiments were performed twice to ensure reproducibility. Data represent average ± s.e.m.

### Testing Reversibility of Splicing Inhibition

Reversibility of inhibition was carried out as previously described,^33^ with the following modifications. Reactions were initiated at high concentration (20 μM) of the tested inhibitor, allowed to proceed for 15 min under splicing conditions (see above), and then diluted by 20-fold with reaction buffer in order to observe whether reaction efficiency increased (as would be the case for a reversible reaction). Aliquots were withdrawn at different time intervals, quenched, and analyzed on an 5% denaturing polyacrylamide gel as previously described.^55^ Experiments were performed in triplicate to ensure reproducibility. Error bars are s.e.m.

### Testing Inhibition of *S. cerevisiae* Growth in YPD and YPG Media

Reduction of yeast growth in the presence of inhibitor molecules was assayed either in liquid YPD media (BD Bacto Yeast Extract, BD Bacto Peptone, 2% glucose) or in YPG media (BD Bacto Yeast Extract, BD Bacto Peptone, 3% glycerol), as previously described^33,34,59^ with the following adaptations. Yeast cultures were grown for 24 h in YPD medium and for 18–20 days in YPG medium. All experiments were performed in triplicate to ensure reproducibility.

### Determination of *C. parapsilosis* MIC Values for Inhibitor Compounds

MICs for *C. parapsilosis* were determined as previously described^33^ according to the guidelines from the protocol M27-A3 from the Clinical and Laboratory Standards Institute (CLSI).^59^ All experiments were performed in triplicate.

### In Cellulo Analysis of Group II Intron Splicing in *C. parapsilosis* by qRT-PCR

*C. parapsilosis* (ATCC: 22019) growth and harvest was performed essentially as described^33^ with the following modifications. Compounds dissolved in DMSO were added to individual cultures to a final concentration of 64 μg/mL. Total RNA was isolated using an E.Z.N.A Fungal RNA Mini Kit (Omega Bio-tek) according to the manufacturer’s protocol. The RNA was eluted in 50 μL of a 1:100 mixture of SUPERaseIn RNase inhibitor (Thermo Fisher): MOPS-EDTA (ME) buffer [10 mM MOPS (pH 6.5), 0.1 mM Na-EDTA (pH 8.5)] followed by DNase treatment with RQ1 DNase (Promega) for 30 min at 37 °C. Then, the reaction was mixed with 7 μL of 3 M NaOAc and precipitated with 80% EtOH at –20 °C. The recovered RNA was resuspended in 30 μL of 1:100 mixture of SUPERaseIn RNase inhibitor/ME buffer. Next, 200 ng of Random Hexamer Primers (Thermo Fisher) was annealed to 10 ng of RNA in 11 μL at 65 °C for 5 min. The annealing reaction mixture was then cooled to RT, followed by the addition of 9 μL of a SuperScript III (Thermo Fisher) master mix: 1X First Strand Synthesis Buffer, 0.5 mM dNTPs (NEB: N0447S), 5 mM DTT, 10U SUPERaseIn RNase inhibitor, and 100 U SuperScript III. Manufacturer reverse transcription conditions were used, followed by an inactivation step at 75 °C for 15 min. After reverse transcription, RNA was degraded by adding 1.5 μL of a 1:1:1 mixture of RNase H (NEB), RNase A (NEB), and RNase T1 (Thermo Fisher) and incubating at 37 °C for 30 min. The cDNA was then purified using AMPure beads (Beckman Coulter) at a 1.8× bead-to-sample ratio according to the manufacturer’s protocol. Purified cDNA was eluted in 30 μL of nuclease-free water. Real-time PCR quantification of cDNA and subsequent determination of the relative levels of *PGK1*, COX1 (total), and COX1 (unspliced) from different conditions were performed in duplicate with three independent samples as previously described.^33^ During analysis, no-RT controls displayed minimal signal comparable to the water-only control, indicating that amplicons had been generated from cDNA and not genomic DNA.

### Cytotoxicity in HEK-293T Cells

For the cytotoxicity experiments, cells were aliquoted into black 96-well plates with a clear bottom (Corning 3603), grown, treated with the inhibitors, and subjected to the Cell Titer Glo cell viability assay (Promega) as previously described.^33^ All experiments were performed in triplicate.

## Supporting Information Available

Structure and activity relationship of quinazoline group II intron inhibitors; synthesis and characterization of quinazoline compounds (PDF)
