# Supplementary material for: Novel Quinazoline Derivatives Inhibit Splicing of Fungal Group II Introns
Source: ACS Chem Biol. 2025 Jan 17;20(2):378–85. doi: 10.1021/acschembio.4c00631 (PMC11851433; doi:10.1021/acschembio.4c00631)
Supplement: Supplementary file 1 — cb4c00631_si_001.pdf [file cb4c00631_si_001.pdf]

Supporting information

Novel quinazoline derivatives inhibit splicing of fungal group II introns.

Olga Fedorova[1,2], Michelle Luo[3], G. Erik Jagdmann Jr.[4,5], Michael C. Van Zandt[4], Luke Sisto[2] and Anna Marie Pyle[1,2,3]*

[1]Howard Hughes Medical Institute.

[2]Department of Molecular, Cellular and Developmental Biology, Yale University, New Haven, CT 06520.

[3]Department of Chemistry, Yale University, New Haven, CT 06520.

[4]New England Discovery Partners, Branford, CT 06405

[5]Present address: ChemoGenics Biopharma, Durham, NC 27707.

*Correspondence to: anna.pyle@yale.edu

Supplementary table 1.

Structure-activity relationships of the quinazoline group II intron inhibitors. Minimal Inhibitory Concentrations (MIC values) for *C. p.* are highlighted in bold.

Compound	Structure	K_i ai5γ intron, μM	MIC *C. parapsilosis,* μg/ml	Internal labeling
**1**	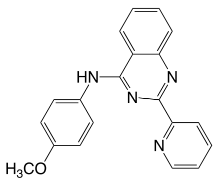	27+6	**16**	NED 55131
2	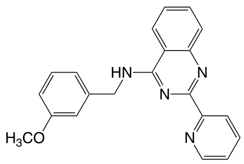	55±19	**>128**	NED 59491
3	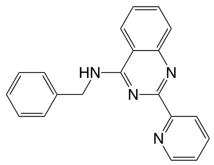	77±25	**8**	NED 59265
4	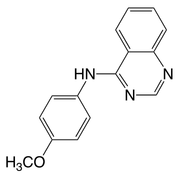	>100	**64**	NED 59396
5	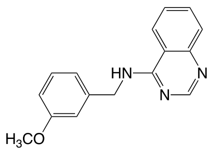	>100	**16**	NED 59395

6	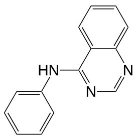	>100	**>128**	NED 59397
7	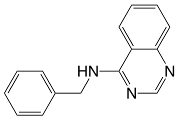	>100	**8**	NED 59399
8	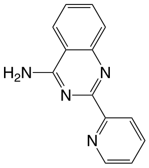	>100	**>128**	NED 59489
9	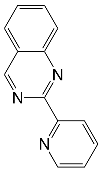	>100	**32**	NED 59501
10	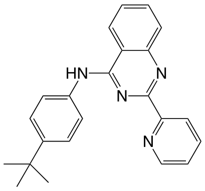	11±2	**8**	NED 55137
11	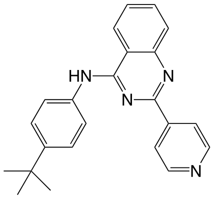	>100	**>128**	NED 55292
12	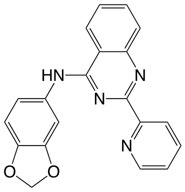	11±2	**>128**	NED 55127

13	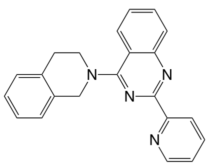	11.4±3.5	**32**	NED 55125
14	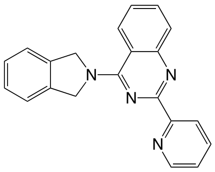	7.6±1.7	**8**	NED 55257
15	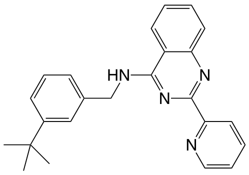	6.4±1.9	**16**	NED 59492
16	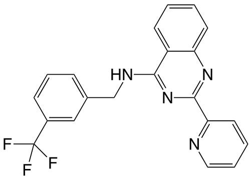	5.6±0.6	**4**	NED 55271
17	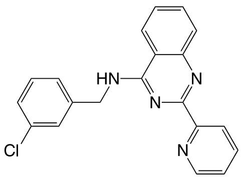	2.6±0.5	**2**	NED 55256
18	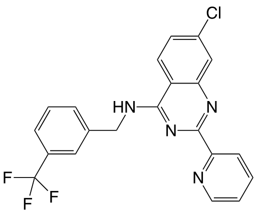	20±6	**>128**	NED 55310
19	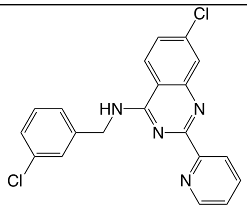	1.4±0.4	**>128**	NED 55309

Materials and Methods

All reactions were performed under a nitrogen atmosphere. Anhydrous solvents were purchased from Sigma-Aldrich® (St. Louis, MO) and Fisher Scientific® (Waltham, MA). Building blocks were obtained from either Combi-Blocks® (San Deigo, CA), Sigma-Aldrich® or TCI® USA (Philadelphia, PA). Palladium catalysts were purchased from TCI®. Additional reagents were sourced from TCI ® and Oakwood Chemical® (Estill, SC). NMR solvents were obtained from Cambridge Isotope Laboratories ® (Tewksbury, MA) and NMR spectra recorded on a Bruker® AdvanceCore 400 MHz spectrometer. HPLC analysis was performed on an Agilent ® 1100 system.

Abbreviations Used

MeCN, acetonitrile

DCM, dichloromethane

DIPEA, N, N-Diisopropylethylamine

equiv., equivalents

Ether, Diethyl ether

EtOAc, ethyl acetate

EtOH, ethanol

ESI, Electrospray Ionization

HPLC, High-Performance Liquid Chromatography

LCMS, Liquid Chromatography Mass Spectrometry

M, molar

MeOH, methanol

NMR, Nuclear Magnetics Resonance

NEt_3, triethylamine

THF, tetrahydrofuran

TLC, thin layer chromatography

pTLC, preparative thin layer chromatography

Synthesis of Common Intermediates

Synthesis of 2-(pyridin-2-yl)quinazolin-4(3H)-one

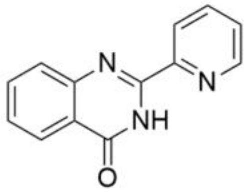

2-(pyridin-2-yl)quinazolin-4(3*H*)-one

A stirred solution of 2-cyanopyridine (2.60 g, 25 mmol) in anhydrous methanol (10 mL) under nitrogen was treated dropwise with a solution of 30% methanolic sodium methoxide (1.13 g, 6.3 mmol) in anhydrous methanol (35 mL), then stirred for 1h. A solution of anthranilic acid (4.29 g, 31.25 mmol) in anhydrous methanol (45 mL) was added over several minutes, then stirred for 45 minutes. The solution was transferred to a pressure bottle, capped, and heated to 65°C overnight. The mixture was cooled to room temperature and about 2/3 of the methanol was removed *in vacuo*. The suspension was cooled on an ice bath for 30 minutes, filtered, and the solid rinsed with cold methanol, collected and dried to afford 4.35 g (78%) of 2-(pyridin-2-yl)quinazolin-4(3H)-one as a white crystalline solid. Procedure adapted from publication (1).

Synthesis of 4-chloro-2-(pyridin-2-yl)quinazoline

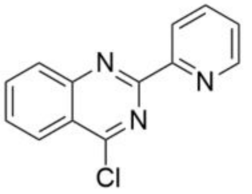

4-chloro-2-(pyridin-2-yl)quinazoline

A mixture of 2-(pyridin-2-yl)quinazolin-4(3H)-one (2.01 g, 9 mmol) and DMF (0.25 mL) was treated with thionyl chloride (40 mL) and heated to 75°C with stirring for 2 h. The solution was cooled and concentrated *in vacuo*, the residual solid was cooled and dissolved in cold water (75 mL). The solution was basified with solid sodium bicarbonate, stirred a few minutes, and filtered. The filtered solid was washed with water and dissolved in DCM, dried (Na_2SO_4) and concentrated *in vacuo*. The crude product was dissolved in ethyl acetate, loaded onto a silica gel column (~200 cc) and eluted with EtOAc, then 5% MeOH in EtOAc to afford 1.99 g (91%) of 4-chloro-2-(pyridin-2-yl)quinazoline as a white solid.

Synthesis of N-(4-(tert-butyl)phenyl)-2-chloroquinazolin-4-amine

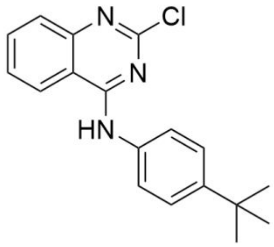

N-(4-(*tert*-butyl)phenyl)-2-chloroquinazolin-4-amine

N-(4-(tert-butyl)phenyl)-2-chloroquinazolin-4-amine was made from 4-(tert-butyl)aniline and 2,4-dichloroquinazoline using a synthetic procedure adapted from patent application (2).

Synthesis and Characterization of Compounds

Compound 1 (N-(4-methoxyphenyl)-2-(pyridin-2-yl)quinazolin-4-amine)

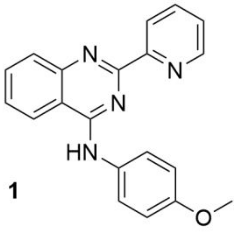

1

4-chloro-2-(pyridin-2-yl)quinazoline (1.0 equiv.) was suspended in 1,4-dioxane to afford a 0.20 M solution and p-anisidine (1.5 equiv.) was added. DIPEA (1.5 equiv.) was added and the reaction was stirred at 110°C for 6 hours. After that time the reaction was cooled and concentrated *in vacuo*. The residue was dissolved in DCM, loaded onto a silica gel column and eluted with DCM-> EtOAc-> 3% MeOH in DCM. The collected solid was recrystallized from MeCN to give **Compound 1** (69%) as a pale-yellow solid.

^{1}H NMR (400 MHz, DMSO-d_6) δ 9.81 (s, 1H), 8.75 (d, *J* = 3.6 Hz, 1H), 8.59 (d, *J* = 8.3 Hz, 1H), 8.36 (d, *J* = 7.9 Hz, 1H), 8.02 – 7.80 (m, 5H), 7.64 (ddd, *J* = 8.2, 6.1, 2.1 Hz, 1H), 7.51 – 7.43 (m, 1H), 7.02 (d, *J* = 9.0 Hz, 2H), 3.79 (s, 3H).

LCMS-ESI m/z calculated for: $C_{20}H_{16}N_4OH^+$ [M+H$^+$] 329.14, found 329.2

HPLC Purity integration at 254 nm = 99.8%

Compound 2 (N-(3-methoxybenzyl)-2-(pyridin-2-yl)quinazolin-4-amine)

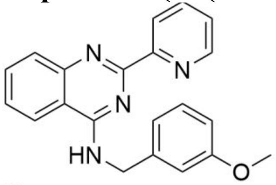

2

4-chloro-2-(pyridin-2-yl)quinazoline (1.0 equiv.) was suspended in EtOH to afford a 0.13 M solution and 3-methoxybenzylamine (1.5 equiv.) was added. DIPEA (2.0 equiv.) was added and the reaction was stirred at 80°C for 2 hours. After that time the reaction was cooled, poured into ice water, and the solid filtered to give **Compound 2** (99%) as a white solid.

^{1}H NMR (400 MHz, $CDCl_3$) δ 8.85 (ddd, *J* = 4.8, 1.9, 0.9 Hz, 1H), 8.59 (dt, *J* = 8.0, 1.1 Hz, 1H), 8.12 (dd, *J* = 8.5, 1.2 Hz, 1H), 7.84 (td, *J* = 7.7, 1.8 Hz, 2H), 7.75 (ddd, *J* = 8.4, 7.0, 1.3 Hz, 1H), 7.47 (ddd, *J* = 8.2, 7.0, 1.2 Hz, 1H), 7.37 (ddd, *J* = 7.5, 4.8, 1.2 Hz, 1H), 7.32 – 7.26 (m, 1H), 7.08 – 7.02 (m, 2H), 6.88 – 6.81 (m, 1H), 6.61 – 6.24 (br, 1H) 5.00 (s, 2H), 3.78 (s, 3H).

LCMS-ESI m/z calculated for $C_{21}H_{18}N_4OH^+$ [M+H$^+$] 343.16, found 343.3

HPLC Purity integration at 254 nm = 100%

Compound 3 (N-benzyl-2-(pyridin-2-yl)quinazolin-4-amine)

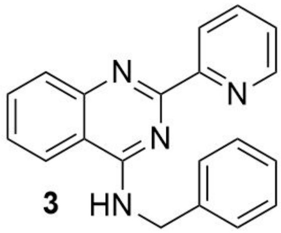

3

4-chloro-2-(pyridin-2-yl)quinazoline (1.0 equiv.) was suspended in EtOH to afford a 0.20 M solution and benzylamine (2.0 equiv.) was added. DIPEA (2.0 equiv.) was added and the reaction was stirred at 80°C for 2 hours. After that time the solvent was removed *in vacuo*, the residue diluted with DCM, and a white solid filtered off. The solid was triturated with ether to give **Compound 3** (77%) as a white powder.

^{1}H NMR (400 MHz, $CDCl_3$) δ 8.87 (ddd, *J* = 4.8, 1.8, 0.9 Hz, 1H), 8.59 (dt, *J* = 7.9, 1.1 Hz, 1H), 8.16 (d, *J* = 8.7 Hz, 1H), 7.83 (td, *J* = 7.7, 1.8 Hz, 1H), 7.79 – 7.71 (m, 2H), 7.52 – 7.42 (m, 3H), 7.41 – 7.29 (m, 4H), 6.09 – 5.96 (br, 1H), 5.03 (d, *J* = 5.3 Hz, 2H).

LCMS-ESI m/z calculated for $C_{20}H_{16}N_4H^+$ [M+H$^+$] 313.14, found 313.65

HPLC Purity integration at 254 nm = 100%

Compound 4 (N-(4-methoxyphenyl)quinazolin-4-amine)

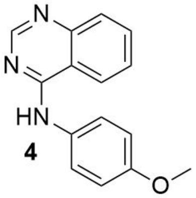

4

4-chloroquinazoline (1.0 equiv.) was suspended in THF to afford a 0.40 M solution and p-anisidine (1.5 equiv.) was added. DIPEA (2.0 equiv.) was added and the reaction was stirred at 22°C for 16 hours. After that time the solvent was removed *in vacuo*, the residue taken up in EtOAc, the organic layer washed with Na_2CO_3, brine, and dried over Na_2SO_4. The mixture was filtered, the solvent removed *in vacuo*, and the residue recrystallized from Ether/MeCN to give **Compound 4** (85%) as a white solid.

^{1}H NMR (400 MHz, MeOD) δ 8.45 (s, 1H), 8.35 (d, *J* = 8.7 Hz, 1H), 7.87 (dd, *J* = 7.0, 1.4 Hz, 1H), 7.85 (dd, *J* = 7.0, 1.4 Hz, 1H), 7.77 (dd, *J* = 8.5, 1.3 Hz, 1H), 7.62 (ddd, *J* = 8.3, 6.9, 1.3 Hz, 1H), 7.60 – 7.53 (m, 2H), 7.07 – 6.91 (m, 2H), 3.83 (s, 3H).

LCMS-ESI m/z calculated for $C_{15}H_{13}N_3OH^+$ [M+H$^+$] 252.11, found 251.9

HPLC Purity integration at 254 nm = 100%

Compound 5 (N-(3-methoxybenzyl)quinazolin-4-amine)

5

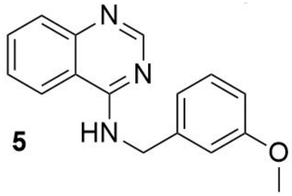

4-chloroquinazoline (1.0 equiv.) was suspended in THF to afford a 0.40 M solution and 3-methoxybenzylamine (1.5 equiv.) was added. NEt_3 (2.0 equiv.) was added and the reaction was stirred at 22°C for 16 hours. After that time the solvent was removed *in vacuo*, the residue taken up in EtOAc, the organic layer washed with Na_2CO_3, brine, and dried over Na_2SO_4. The mixture was filtered, the solvent removed *in vacuo*, and the residue recrystallized from Ether/MeCN to give **Compound 5** (59%) as a white solid.

^{1}H NMR (400 MHz, MeOD) δ 8.42 (s, 1H), 8.13 (dd, *J* = 8.4, 0.8 Hz, 1H), 7.75 (ddd, *J* = 8.3, 6.9, 1.3 Hz, 1H), 7.71 – 7.66 (m, 1H), 7.49 (ddd, *J* = 8.3, 6.8, 1.4 Hz, 1H), 7.19 (t, *J* = 8.1 Hz, 1H), 6.98 – 6.87 (m, 2H), 6.77 (ddd, *J* = 8.3, 2.6, 1.0 Hz, 1H), 4.81 (s, 2H), 3.72 (s, 3H).

LCMS-ESI m/z calculated for $C_{16}H_{15}N_3OH^+$ [M+H$^+$] 266.13, found 266.1

HPLC Purity integration at 254 nm = 100%

Compound 6 (N-phenylquinazolin-4-amine)

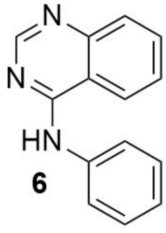

4-chloroquinazoline (1.0 equiv.) was suspended in THF to afford a 0.24 M solution and aniline (1.5 equiv.) was added. NEt_3 (2.0 equiv) was added and the reaction was stirred at 22°C for 16 hours. After that time the solvent was removed *in vacuo*, the residue taken up in EtOAc, the organic layer washed with Na_2CO_3, brine, and dried over Na_2SO_4. The mixture was filtered, the solvent removed *in vacuo*, and the residue recrystallized from DCM to give **Compound 6** (78%) as a white solid.

^{1}H NMR (400 MHz, MeOD) δ 8.72 (s, 1H), 8.61 (d, J = 8.3 Hz, 1H), 8.07 (t, J = 7.8 Hz, 1H), 7.84 (t, J = 8.4 Hz, 2H), 7.73 (d, J = 7.9 Hz, 2H), 7.48 (t, J = 7.8 Hz, 2H), 7.34 (t, J = 7.5 Hz, 1H).

LCMS-ESI m/z calculated for $C_{14}H_{11}N_3H^+$ [M+H$^+$] 222.09, found 222.1

HPLC Purity integration at 254 nm = 100%

Compound 7 (N-benzylquinazolin-4-amine)

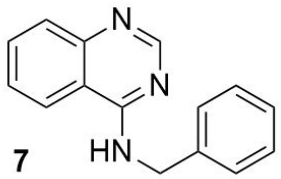

4-chloroquinazoline (1.0 equiv.) was suspended in THF to afford a 0.36M solution and benzylamine (1.5 equiv.) was added. NEt_3 (3.0 equiv) was added and the reaction was stirred at 22°C for 16 hours. After that time the solvent was removed *in vacuo*, the residue taken up in EtOAc, the organic layer washed with Na_2CO_3, brine, and dried over Na_2SO_4. The mixture was filtered, the solvent removed *in vacuo*, and the residue recrystallized from Ether to give **Compound 7** (61%) as a white solid.

^{1}H NMR (400 MHz, MeOD) δ 8.43 (s, 1H), 8.15 (dd, J = 8.4, 1.3 Hz, 1H), 7.78 (ddd, J = 8.3, 6.9, 1.4 Hz, 1H), 7.71 (dd, J = 8.3, 1.4 Hz, 1H), 7.52 (ddd, J = 8.3, 6.9, 1.4 Hz, 1H), 7.42 – 7.34 (m, 2H), 7.35 – 7.26 (m, 2H), 7.26 – 7.16 (m, 1H), 4.85 (s, 2H).

LCMS-ESI m/z calculated for $C_{15}H_{13}N_3H^+$ [M+H$^+$] 236.12, found 235.9

HPLC Purity integration at 254 nm = 100%

Compound 8 (2-(pyridin-2-yl)quinazolin-4-amine) & 8S (4-methoxy-2-(pyridin-2-yl)quinazoline)

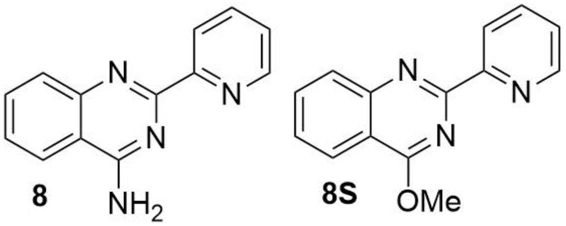

8 8S

4-chloro-2-(pyridin-2-yl)quinazoline (1.0 equiv.) was dissolved in 7N methanolic ammonia to afford a 0.24 M solution. The reaction was stirred at 22°C for 16 hours. The mixture was concentrated in vacuo and the residue taken up in 1N aqueous sodium hydroxide. This was extracted with 9:1 DCM/isopropanol and the combined organic solution was washed with brine, dried over Na_2SO_4, filtered, and concentrated *in vacuo*. The residue was dissolved in DCM, loaded onto a silica gel column and eluted with 50% EtOAc in DCM to afford 4-methoxy-2-(pyridin-2-yl)quinazoline (**Compound 8S**, 44%) as a white solid, then with 10% MeOH in DCM to afford 2-(pyridin-2-yl)quinazolin-4-amine (**Compound 8**, 39%) as a white solid.

Compound 8 Characterization

^{1}H NMR (400 MHz, $CDCl_3$) δ 8.78 (s, 1H), 8.56 (d, *J* = 7.9 Hz, 1H), 8.09 (d, *J* = 8.3 Hz, 1H), 8.00 (d, *J* = 8.2 Hz, 1H), 7.93 – 7.69 (m, 2H), 7.61 – 7.29 (m, 2H).

LCMS-ESI m/z calculated for: $C_{13}H_{10}N_4H^+$ [M+H$^+$] 223.10, found 223.2

HPLC Purity integration at 254 nm = 97.8%

Compound 8S Characterization

^{1}H NMR (400 MHz, $CDCl_3$) δ 8.95 (s, 1H), 8.67 (d, *J* = 8.0 Hz, 1H), 8.20 (d, *J* = 8.4 Hz, 2H), 7.90 (m, 2H), 7.59 (t, *J* = 7.6 Hz, 1H), 7.45 (t, *J* = 6.2 Hz, 1H), 4.35 (s, 3H).

LCMS-ESI m/z calculated for: $C_{14}H_{11}N_3OH^+$ [M+H$^+$] 238.10, found 238.2

HPLC Purity integration at 254 nm = 98.4%

Compound 9 (2-(pyridin-2-yl)quinazoline)

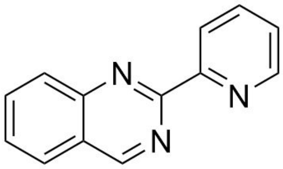

9

Copper iodide (15 mol%), cesium carbonate (3.0 equiv.), picolinimidamide hydrochloride (1.5 equiv.), and iodobenzaldehyde (1.0 equiv.) were added to a pressure tube, followed by methanol to make a 0.40 M solution. The tube was purged with nitrogen and heated to 60°C for 16 hours. The reaction mixture was cooled to room temperature. Water and EtOAc were added to the reaction mixture and stirred thoroughly. The mixture was filtered through Celite®, washed 3 times with EtOAc, and concentrated *in vacuo*. The residue was purified by flash column chromatography over silica gel using 2-5% MeOH in EtOAc. **Compound 9** (56%) was obtained as a beige solid.

^{1}H NMR (400 MHz, $CDCl_3$) δ 9.54 (s, 1H), 8.88 (ddd, *J* = 4.8, 1.8, 0.9 Hz, 1H), 8.67 (dt, *J* = 8.0, 1.1 Hz, 1H), 8.22 (dd, *J* = 8.5, 1.1 Hz, 1H), 7.98 – 7.84 (m, 3H), 7.64 (ddd, *J* = 8.1, 6.9, 1.1 Hz, 1H), 7.39 (ddd, *J* = 7.5, 4.7, 1.2 Hz, 1H).

LCMS-ESI m/z calculated for $C_{13}H_9N_3H^+$ [M+H$^+$] 208.09, found 208.4

HPLC Purity integration at 254 nm = 100%

Compound 10 (N-(4-(tert-butyl)phenyl)-2-(pyridin-2-yl)quinazolin-4-amine)

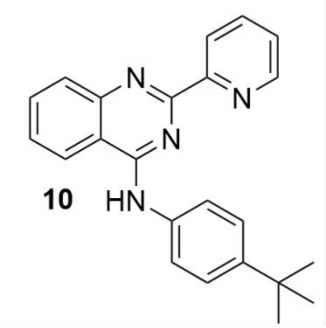

4-chloro-2-(pyridin-2-yl)quinazoline (1.0 equiv.) was suspended in 1,4-dioxane to afford a 0.05 M solution and 4-tert-butylaniline (1.5 equiv.) was added. DIPEA (1.5 equiv.) was added and the reaction was stirred at 110°C for 6 hours. After that time the reaction was cooled and concentrated *in vacuo*. The residue was dissolved in DCM and purified by pTLC (3% MeOH in DCM) to give **Compound 10** (65%) as a white solid.

^{1}H NMR (400 MHz, DMSO-d_6) δ 9.82 (s, 1H), 8.77 (d, *J* = 3.6 Hz, 1H), 8.63 (d, *J* = 8.3 Hz, 1H), 8.40 (d, *J* = 7.9 Hz, 1H), 8.05 (d, *J* = 8.5 Hz, 2H), 7.98 (td, *J* = 7.7, 1.8 Hz, 1H), 7.94 – 7.84 (m, 2H), 7.66 (ddd, *J* = 8.2, 6.0, 2.1 Hz, 1H), 7.50 (ddd, *J* = 7.5, 4.7, 1.2 Hz, 1H), 7.45 (d, *J* = 8.7 Hz, 2H), 1.33 (s, 9H).

LCMS-ESI m/z calculated for: $C_{23}H_{22}N_4H^+$ [M+H$^+$] 355.19, found 355.2

HPLC Purity integration at 254 nm = 100%

Compound 11 (N-(4-(tert-butyl)phenyl)-2-(pyridin-4-yl)quinazolin-4-amine)

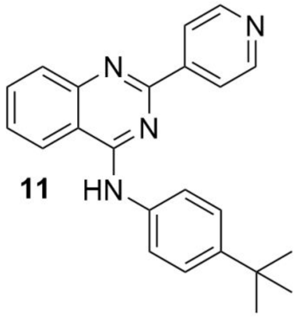

11

N-(4-(tert-butyl)phenyl)-2-chloroquinazolin-4-amine (1.0 equiv.), pyridine-4-boronic acid (1.5 equiv.), and K_2CO_3 (5.0 equiv.) were dissolved in 2:1 1,4-dioxane:water to afford a 0.10 M solution. The solution was rigorously sparged with argon and Pd(dppf)Cl_2.CH_2Cl_2 (10 mol%) was added before stirring at 90°C for 4 hours. The reaction was then cooled, diluted with DCM, dried over Na_2SO_4, filtered, and the solvent removed *in vacuo*. The residue was dissolved in DCM and purified by pTLC (20% Hexane in EtOAc) to give **Compound 11** (83%) as a white solid.

^{1}H NMR (400 MHz, DMSO-d_6) δ 9.93 (s, 1H), 8.76 (dd, *J* = 4.5, 1.6 Hz, 2H), 8.64 (d, *J* = 8.3 Hz, 1H), 8.29 (dd, *J* = 4.5, 1.6 Hz, 2H), 7.94 – 7.90 (m, 3H), 7.68 (dt, *J* = 8.3, 4.1 Hz, 1H), 7.53 – 7.45 (m, 3H), 1.34 (s, 9H).

LCMS-ESI m/z calculated for: $C_{23}H_{22}N_4H^+$ [M+H$^+$] 355.19, found 355.3

HPLC Purity integration at 254 nm = 97.4%

Compound 12 (N-(benzo[d][1,3]dioxol-5-yl)-2-(pyridin-2-yl)quinazolin-4-amine)

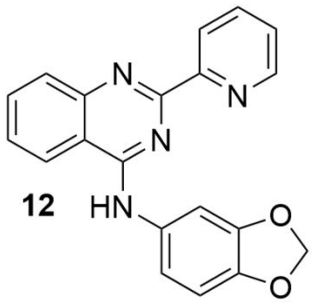

12

4-chloro-2-(pyridin-2-yl)quinazoline (1.0 equiv.) was suspended in 1,4-dioxane to afford a 0.20 M solution and 1,3-benzodioxol-5-amine (1.5 equiv.) was added. DIPEA (1.5 equiv.) was added and the reaction was stirred at 110°C for 6 hours. After that time the reaction was cooled and concentrated *in vacuo*. The residue was dissolved in DCM and purified by pTLC (3% MeOH in DCM) to give **Compound 12** (53%) as a white solid.

^{1}H NMR (400 MHz, DMSO-d_6) δ 9.80 (s, 1H), 8.75 (ddd, *J* = 4.7, 1.9, 0.9 Hz, 1H), 8.58 (d, *J* = 8.3 Hz, 1H), 8.37 (dt, *J* = 7.9, 1.1 Hz, 1H), 8.02 – 7.84 (m, 4H), 7.65 (ddd, *J* = 8.3, 5.9, 2.3 Hz, 1H), 7.50 (ddd, *J* = 7.6, 4.7, 1.2 Hz, 1H), 7.41 (dd, *J* = 8.5, 2.1 Hz, 1H), 6.98 (d, *J* = 8.4 Hz, 1H), 6.06 (s, 2H).

LCMS-ESI m/z calculated for: $C_{20}H_{14}N_4O_2H^+$ [M+H$^+$] 343.12, found 343.2

HPLC Purity integration at 254 nm = 97.4%

Compound 13 (4-(3,4-dihydroisoquinolin-2(1H)-yl)-2-(pyridin-2-yl)quinazoline)

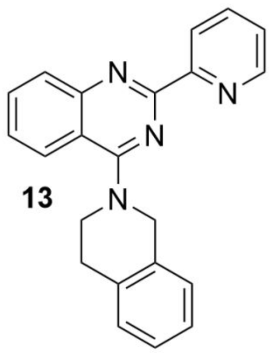

4-chloro-2-(pyridin-2-yl)quinazoline (1.0 equiv.) was suspended in EtOH to afford a 0.20 M solution and 1,2,3,4-tetrahydroisoquinoline (1.5 equiv.) was added. DIPEA (1.5 equiv.) was added and the reaction was stirred at 80°C for 2 hours. After that time the reaction was cooled, poured into ice water, and **Compound 13** (89%) was filtered off as a beige solid.

^{1}**H NMR** (400 MHz, DMSO-d_6) δ 8.77 (ddd, *J* = 4.7, 1.8, 0.9 Hz, 1H), 8.51 (dt, *J* = 7.9, 1.1 Hz, 1H), 8.18 (dd, *J* = 8.4, 1.4 Hz, 1H), 7.98 (td, *J* = 7.7, 1.8 Hz, 1H), 7.96 – 7.91 (m, 1H), 7.87 (ddd, *J* = 8.3, 6.8, 1.3 Hz, 1H), 7.60 (ddd, *J* = 8.3, 6.8, 1.4 Hz, 1H), 7.51 (ddd, *J* = 7.5, 4.7, 1.2 Hz, 1H), 7.31 (dd, *J* = 5.3, 3.7 Hz, 1H), 7.28 – 7.19 (m, 3H), 5.04 (s, 2H), 4.10 (t, *J* = 5.8 Hz, 2H), 3.17 (t, *J* = 5.8 Hz, 2H).

LCMS-ESI m/z calculated for: $C_{22}H_{18}N_4H^+$ [M+H$^+$] 339.16, found 339.3

HPLC Purity integration at 254 nm = 97.3%

Compound 14 (4-(isoindolin-2-yl)-2-(pyridin-2-yl)quinazoline)

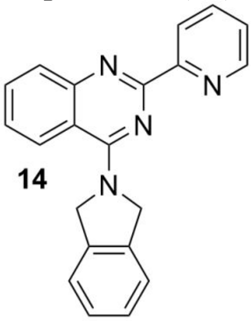

14

4-chloro-2-(pyridin-2-yl)quinazoline (1.0 equiv.) was suspended in EtOH to afford a 0.20 M solution and isoindoline (1.5 equiv.) was added. DIPEA (1.5 equiv.) was added and the reaction was stirred at 80°C for 2 hours. After that time the reaction was cooled, poured into ice water, and **Compound 14** (48%) was filtered off as a beige solid.

^{1}H NMR (400 MHz, DMSO-d_6) δ 8.76 (ddd, *J* = 4.7, 1.8, 0.9 Hz, 1H), 8.60 (d, *J* = 8.5 Hz, 1H), 8.54 (d, *J* = 7.8 Hz, 1H), 7.98 (td, *J* = 7.7, 1.8 Hz, 1H), 7.91 (dd, *J* = 8.3, 1.5 Hz, 1H), 7.85 (ddd, *J* = 8.2, 6.7, 1.1 Hz, 1H), 7.58 (ddd, *J* = 8.4, 6.7, 1.5 Hz, 1H), 7.54 – 7.48 (m, 3H), 7.37 (dd, *J* = 5.6, 3.1 Hz, 2H), 5.46 (s, 4H).

LCMS-ESI m/z calculated for: $C_{21}H_{16}N_4H^+$ [M+H$^+$] 325.14, found 324.8

HPLC Purity integration at 254 nm = 100%

Compound 15 (N-(3-(tert-butyl)benzyl)-2-(pyridin-2-yl)quinazolin-4-amine)

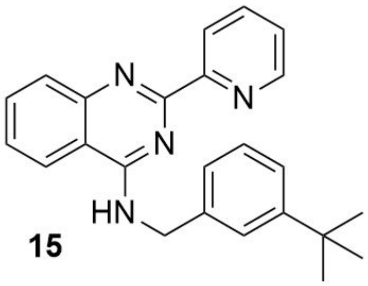

15

4-chloro-2-(pyridin-2-yl)quinazoline (1.0 equiv.) was suspended in EtOH to afford a 0.13 M solution and (3-(tert-butyl)phenyl)methanamine (1.5 equiv.) was added. DIPEA (2.0 equiv.) was added and the reaction was stirred at 80°C for 2 hours. After that time the reaction was cooled, poured into ice water, and the crude product filtered off. The crude product was purified by flash column chromatography over silica gel using 5% MeOH in DCM. **Compound 15** (56%) was obtained as a white solid.

^{1}H NMR (400 MHz, $CDCl_3$) δ 8.78 (ddd, *J* = 4.8, 1.8, 0.9 Hz, 1H), 8.54 (dt, *J* = 8.0, 1.1 Hz, 1H), 8.06 (dd, *J* = 8.8, 0.9 Hz, 1H), 7.75 (td, *J* = 7.7, 1.8 Hz, 1H), 7.71 – 7.60 (m, 2H), 7.46 – 7.41 (m, 1H), 7.37 (ddd, *J* = 8.3, 7.0, 1.2 Hz, 1H), 7.32 – 7.15 (m, 4H), 6.29 – 5.99 (br, 1H) 4.92 (d, *J* = 2.5 Hz, 2H), 1.24 (s, 9H).

LCMS-ESI m/z calculated for $C_{24}H_{24}N_4H^+$ [M+H$^+$] 369.21, found 369.4

HPLC Purity integration at 254 nm = 97.2%

Compound 16 (2-(pyridin-2-yl)-N-(3-(trifluoromethyl)benzyl)quinazolin-4-amine)

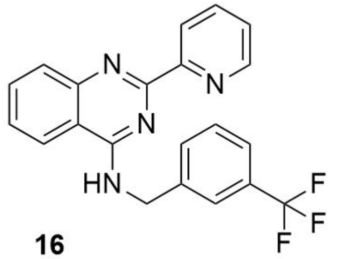

16

4-chloro-2-(pyridin-2-yl)quinazoline (1.0 equiv.) was suspended in EtOH to afford a 0.20 M solution and 3-(trifluoromethyl)benzylamine (1.5 equiv.) was added. DIPEA (1.5 equiv.) was added and the reaction was stirred at 80°C for 2 hours. After that time the reaction was cooled, poured into ice water, and **Compound 16** (51%) was filtered off as a beige solid.

^{1}**H NMR** (400 MHz, DMSO-d$_6$) δ 9.04 (t, *J* = 5.9 Hz, 1H), 8.72 (ddd, *J* = 4.7, 1.9, 0.9 Hz, 1H), 8.35 (dt, *J* = 7.9, 1.1 Hz, 1H), 8.32 (dd, *J* = 8.3, 1.2 Hz, 1H), 7.96 (s, 1H), 7.89 (td, *J* = 7.7, 1.8 Hz, 1H), 7.85 – 7.82 (m, 2H), 7.79 (d, *J* = 7.3 Hz, 1H), 7.63 – 7.51 (m, 3H), 7.47 (ddd, *J* = 7.5, 4.7, 1.2 Hz, 1H), 4.96 (d, *J* = 5.6 Hz, 2H).

LCMS-ESI m/z calculated for: $C_{21}H_{15}F_3N_4H^+$ [M+H$^+$] 381.13, found 380.8

HPLC Purity integration at 254 nm = 97.8%

Compound 17 (N-(3-chlorobenzyl)-2-(pyridin-2-yl)quinazolin-4-amine)

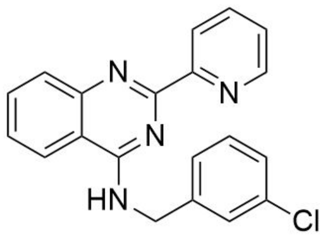

17

4-chloro-2-(pyridin-2-yl)quinazoline (1.0 equiv.) was suspended in EtOH to afford a 0.20 M solution and 3-chlorobenzylamine (1.5 equiv.) was added. DIPEA (1.5 equiv.) was added and the reaction was stirred at 80°C for 2 hours. After that time the reaction was cooled, poured into ice water, and **Compound 17 (**68%) was filtered off as a brown solid.

^{1}H NMR (400 MHz, DMSO-d_6) δ 8.97 (t, *J* = 5.9 Hz, 1H), 8.73 (ddd, *J* = 4.7, 1.8, 0.9 Hz, 1H), 8.35 (dd, *J* = 18.6, 8.1 Hz, 2H), 7.91 (td, *J* = 7.7, 1.8 Hz, 1H), 7.86 – 7.79 (m, 2H), 7.62 – 7.57 (d, *J* = 1.8 Hz, 1H), 7.57 (ddd, *J* = 8.2, 5.7, 2.4 Hz, 1H), 7.50 – 7.46 (m, 1H), 7.46 – 7.41 (m, 1H), 7.35 (t, *J* = 7.7 Hz, 1H), 7.32 – 7.26 (m, 1H), 4.90 (d, *J* = 5.7 Hz, 2H).

LCMS-ESI m/z calculated for: $C_{20}H_{15}ClN_4H^+$ [M+H$^+$] 347.11, found 346.7

HPLC Purity integration at 254 nm = 98.4%

Compound 18 (7-chloro-2-(pyridin-2-yl)-N-(3-(trifluoromethyl)benzyl)quinazolin-4-amine)

18

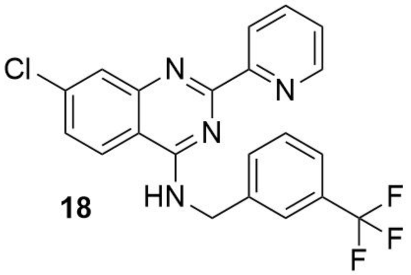

4,7-dichloro-2-(pyridin-2-yl)quinazoline (1.0 equiv.) was suspended in EtOH to afford a 0.18M solution and 3-(trifluoromethyl)benzylamine (1.5 equiv.) was added. DIPEA (1.5 equiv.) was added and the reaction was stirred at 80°C for 2 hours. After that time the reaction was poured into ice water and extracted with EtOAc. The organic layer was washed with brine, dried over Na_2SO_4, and filtered. The solvent was removed *in vacuo* and the solid was triturated by stirring in ether to give **Compound 18** (30%) as a white solid.

^{1}H NMR (400 MHz, DMSO-d_6) δ 9.17 (t, *J* = 5.9 Hz, 1H), 8.73 (d, *J* = 4.3 Hz, 1H), 8.35 (dd, *J* = 8.5, 3.4 Hz, 2H), 8.03 – 7.83 (m, 3H), 7.78 (d, *J* = 7.4 Hz, 1H), 7.66 – 7.52 (m, 3H), 7.48 (ddd, *J* = 7.5, 4.7, 1.2 Hz, 1H), 4.95 (d, *J* = 5.6 Hz, 2H).

LCMS-ESI m/z calculated for: $C_{21}H_{14}ClF_3N_4H^+$ [M+H$^+$] 415.09, found 415.3

HPLC Purity integration at 254 nm = 100%

Compound 19 (7-chloro-N-(3-chlorobenzyl)-2-(pyridin-2-yl)quinazolin-4-amine)

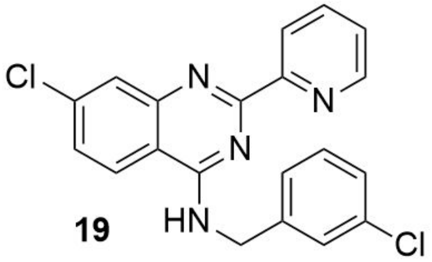

19

4,7-dichloro-2-(pyridin-2-yl)quinazoline (1.0 equiv.) was suspended in EtOH to afford a 0.18 M solution and 3-chlorobenzylamine (1.5 equiv.) was added. DIPEA (1.5 equiv.) was added and the reaction was stirred at 80°C for 2 hours. After that time the reaction was poured into ice water and extracted with EtOAc. The organic layer was washed with brine, dried over Na_2SO_4, and filtered. The solvent was removed *in vacuo* and the solid was triturated by stirring in ether to give **Compound 19** (48%) as a white solid.

^{1}H NMR (400 MHz, DMSO-d_6) δ 9.10 (t, *J* = 5.9 Hz, 1H), 8.74 (dd, *J* = 5.0, 1.6 Hz, 1H), 8.36 (dd, *J* = 8.5, 2.4 Hz, 2H), 7.92 (td, *J* = 7.7, 1.8 Hz, 1H), 7.87 (d, *J* = 2.1 Hz, 1H), 7.64 – 7.57 (m, 2H), 7.48 (ddd, *J* = 7.5, 4.7, 1.2 Hz, 1H), 7.43 (d, *J* = 7.6 Hz, 1H), 7.35 (t, *J* = 7.7 Hz, 1H), 7.32 – 7.27 (m, 1H), 4.89 (d, *J* = 5.5 Hz, 2H).

LCMS-ESI m/z calculated for: $C_{20}H_{14}Cl_2N_4H^+$ [M+H$^+$] 381.07, found 381.2

HPLC Purity integration at 254 nm = 100%

Characterization Data for Representative Compounds

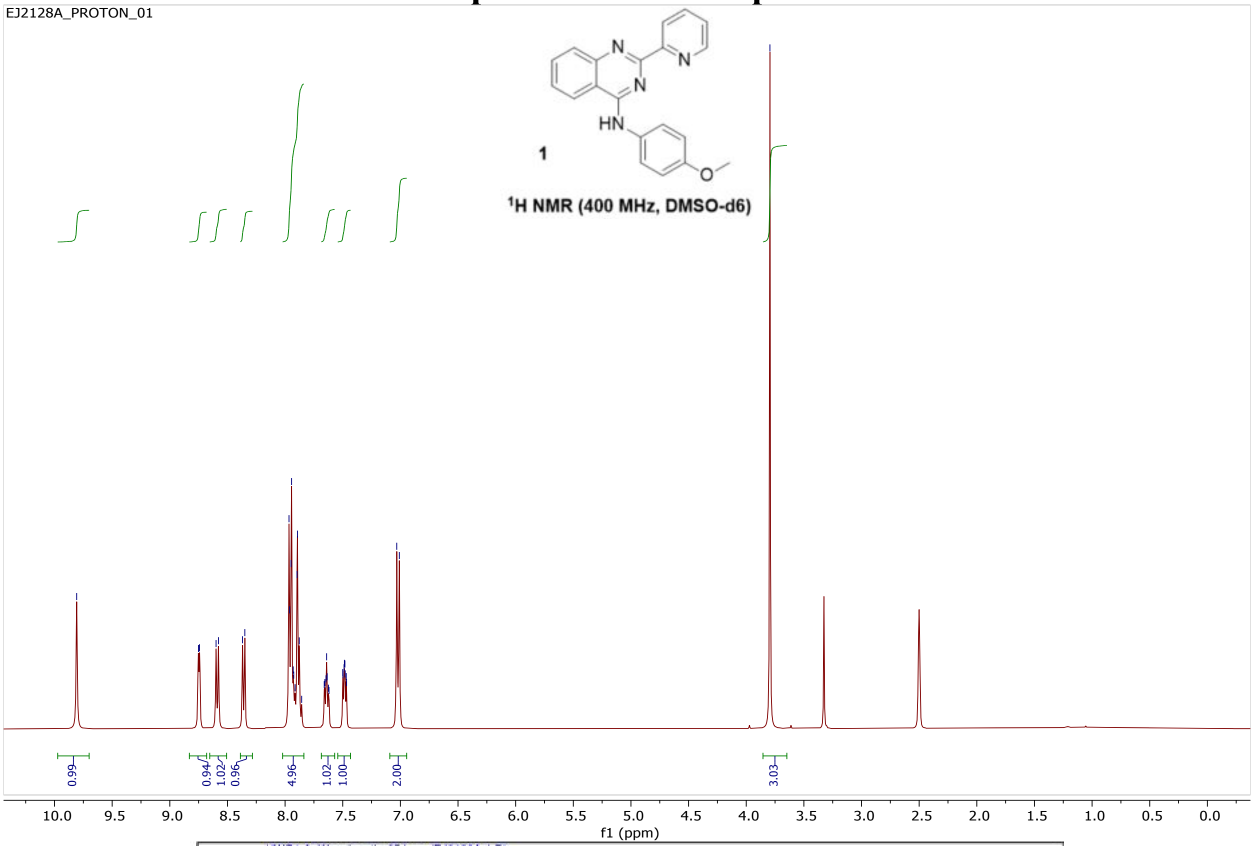

1 HPLC

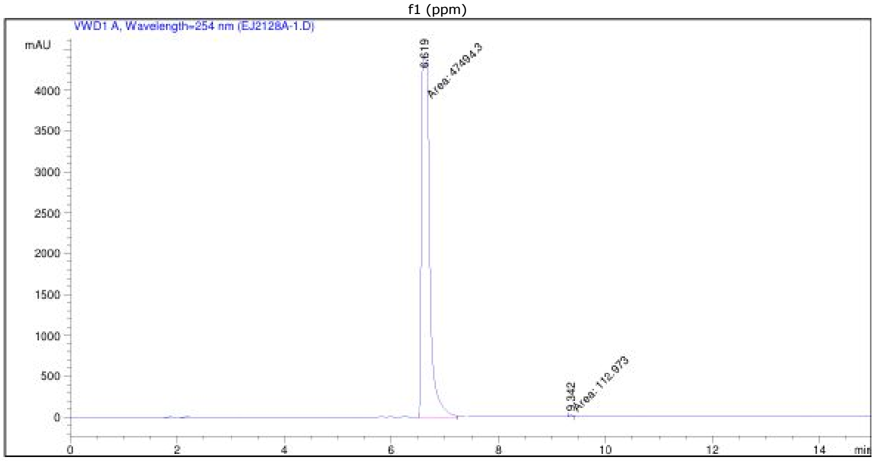


```
=====================================================================
                        Area Percent Report
=====================================================================

Sorted By             :      Signal
Multiplier            :      1.0000
Dilution              :      1.0000
Use Multiplier & Dilution Factor with ISTDs


Signal 1: VWD1 A, Wavelength=254 nm

Peak RetTime Type  Width     Area      Height      Area
  #   [min]        [min]  mAU   *s    [mAU   ]       %
----|-------|----|-------|----------|----------|--------|
   1   6.619 MM    0.1792 4.74943e4  4418.34229  99.7627
   2   9.342 MM    0.0672  112.97293   28.02481   0.2373

Totals :                  4.76072e4  4446.36710

=====================================================================
                          *** End of Report ***
```

1 LCMS

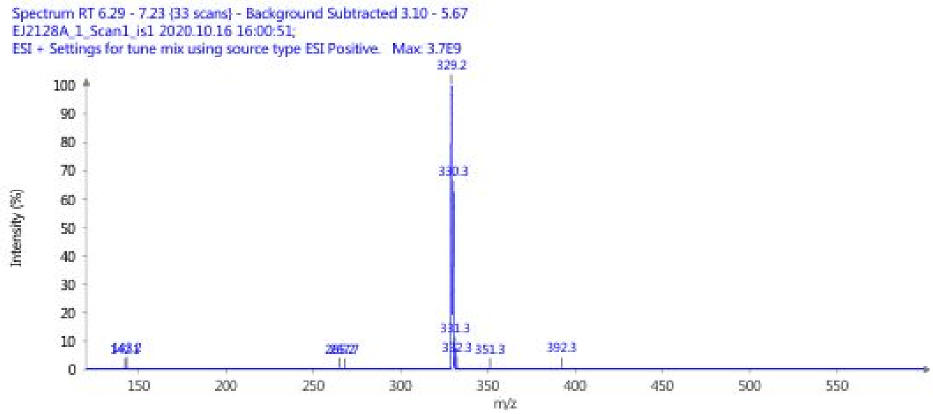
Spectrum RT 6.29 - 7.23 (33 scans) - Background Subtracted 3.10 - 5.67
EJ2128A_1_Scan1_is1 2020.10.16 16:00:51;
ESI + Settings for tune mix using source type ESI Positive. Max: 3.7E9
329.2
330.3
331.3
351.3
392.3
Intensity (%)
m/z

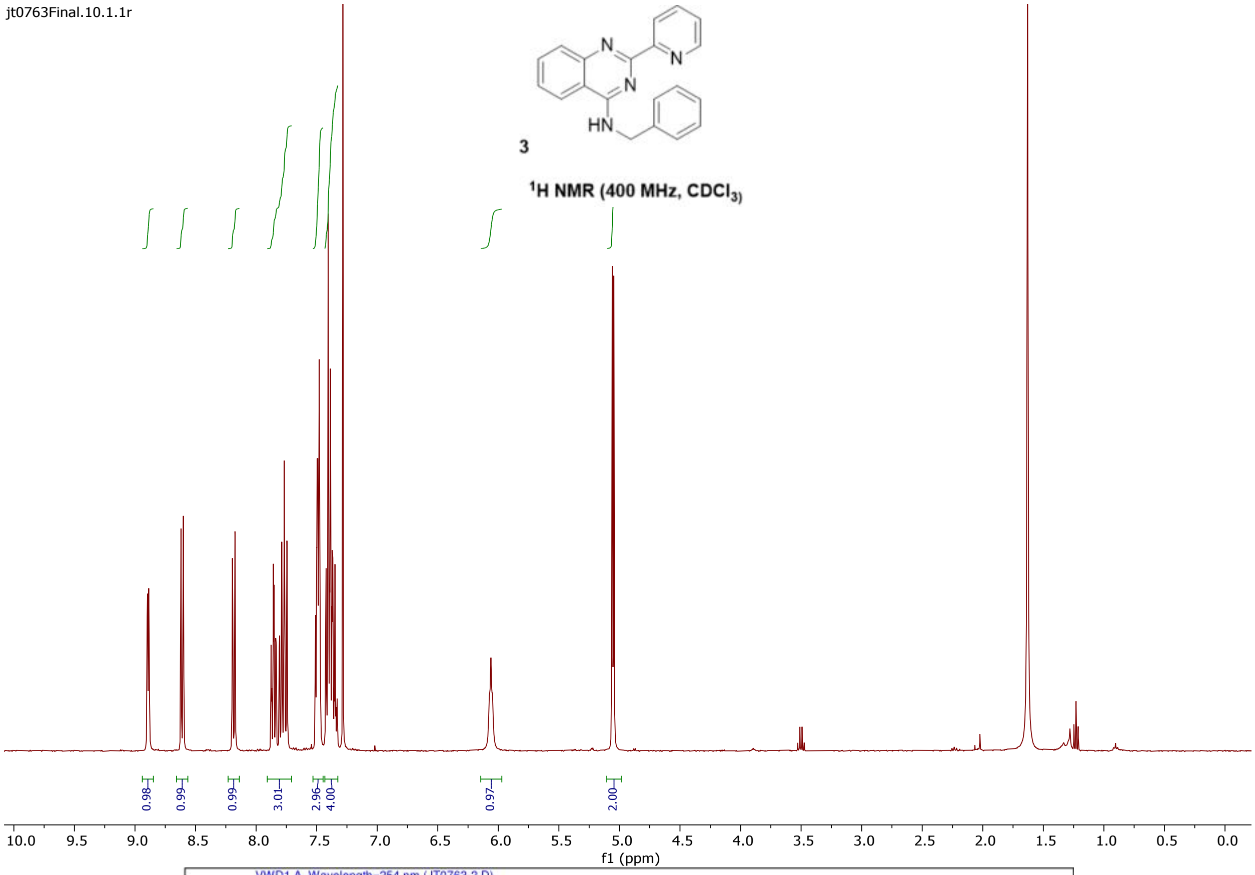

3 HPLC

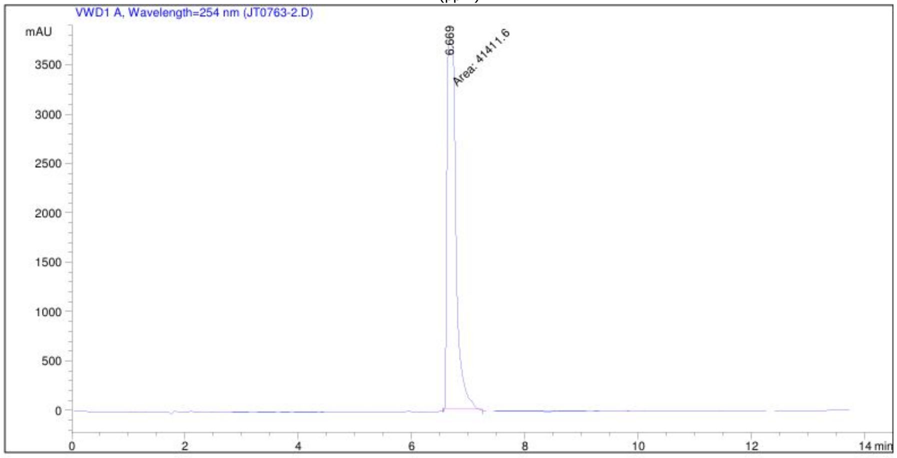


```
=====================================================================
                         Area Percent Report
=====================================================================

Sorted By             :      Signal
Multiplier            :      1.0000
Dilution              :      1.0000
Use Multiplier & Dilution Factor with ISTDs


Signal 1: VWD1 A, Wavelength=254 nm

Peak RetTime Type  Width     Area      Height     Area
  #   [min]        [min]  mAU   *s   [mAU   ]      %
----|-------|----|-------|----------|----------|--------|
   1   6.669 MM    0.1863 4.14116e4  3703.83398 100.0000

Totals :                  4.14116e4  3703.83398


=====================================================================
                          *** End of Report ***
```

3 LCMS

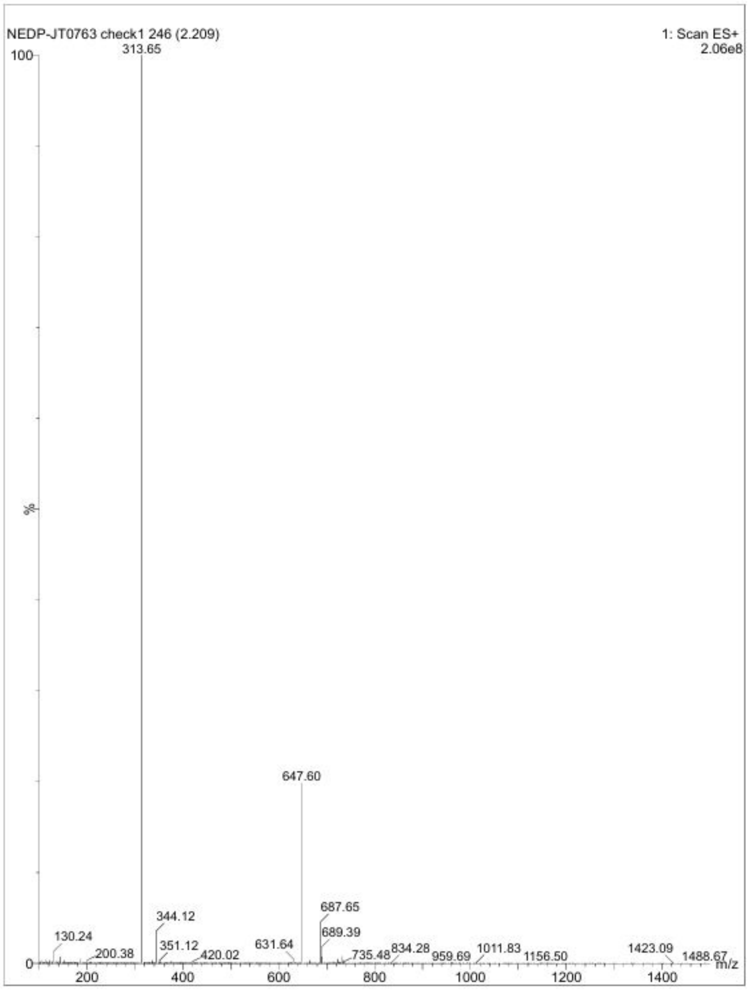
NEDP-JT0763 check1 246 (2.209)
1: Scan ES+
2.06e8
100
%
0
313.65
647.60
687.65
689.39
344.12
351.12
130.24
200.38
420.02
631.64
735.48
834.28
959.69
1011.83
1156.50
1423.09
1488.67
m/z
200
400
600
800
1000
1200
1400

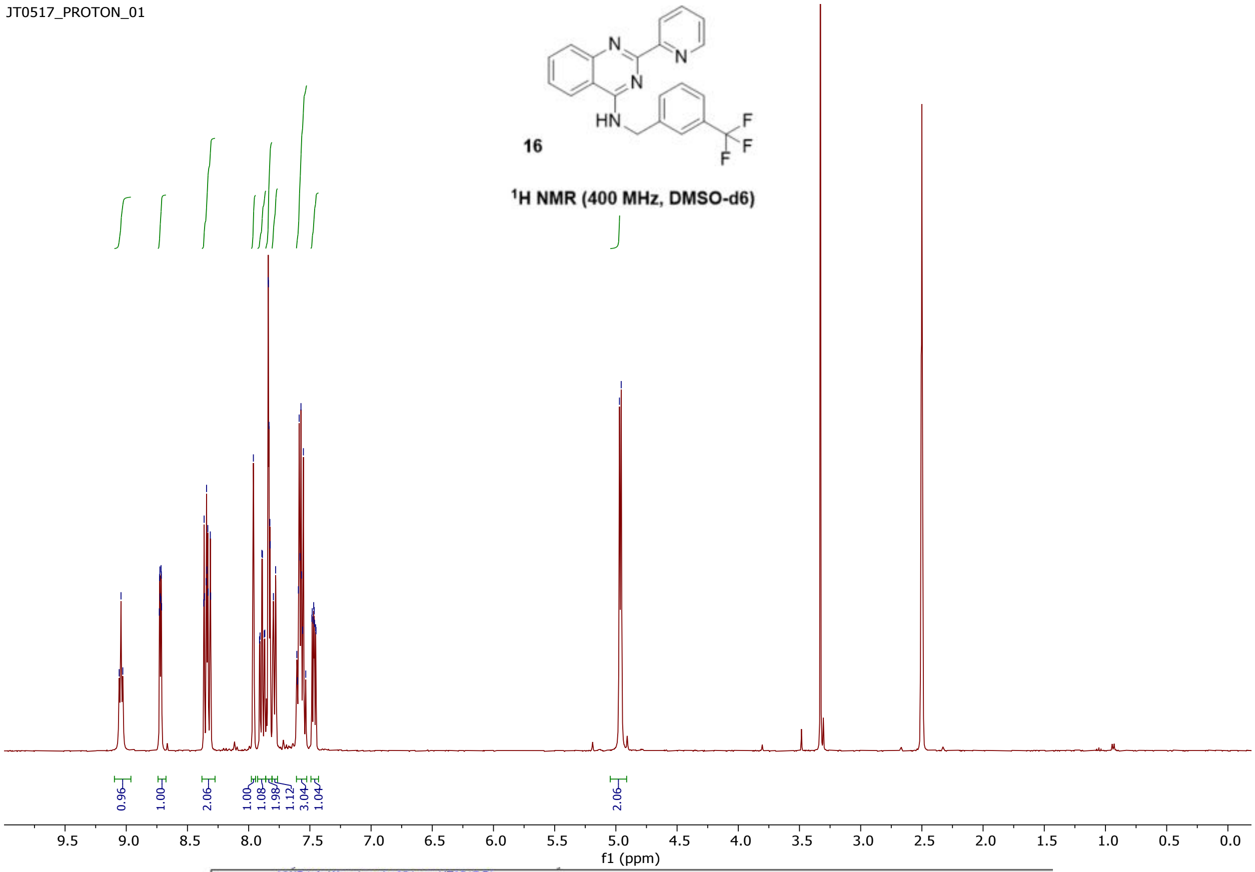

16 HPLC

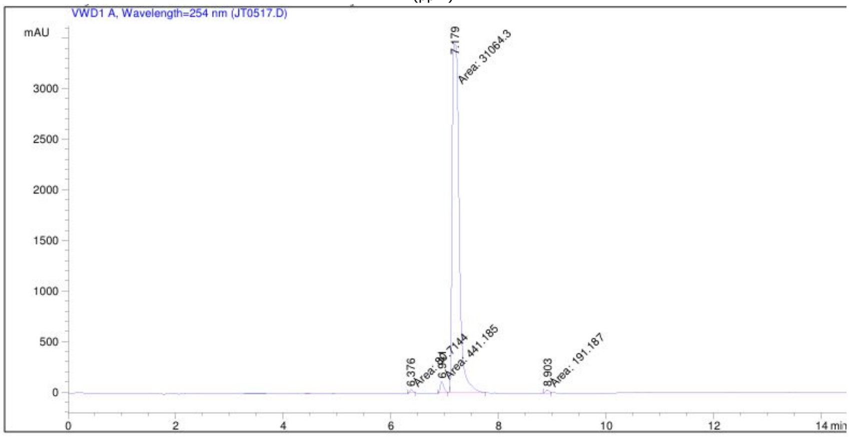


```
=====================================================================
                         Area Percent Report
=====================================================================

Sorted By             :      Signal
Multiplier            :      1.0000
Dilution              :      1.0000
Use Multiplier & Dilution Factor with ISTDs


Signal 1: VWD1 A, Wavelength=254 nm

Peak RetTime Type  Width     Area      Height     Area
  #   [min]        [min]  mAU   *s   [mAU    ]     %
----|-------|----|-------|----------|----------|--------|
   1   6.376 MM    0.0532   80.71436   25.30557   0.2540
   2   6.941 MM    0.0701  441.18497  104.87093   1.3884
   3   7.179 MM    0.1502 3.10643e4  3447.37012  97.7560
   4   8.903 MM    0.0852  191.18700   37.40136   0.6016

Totals :                  3.17774e4  3614.94798


=====================================================================
                          *** End of Report ***
```

16 LCMS

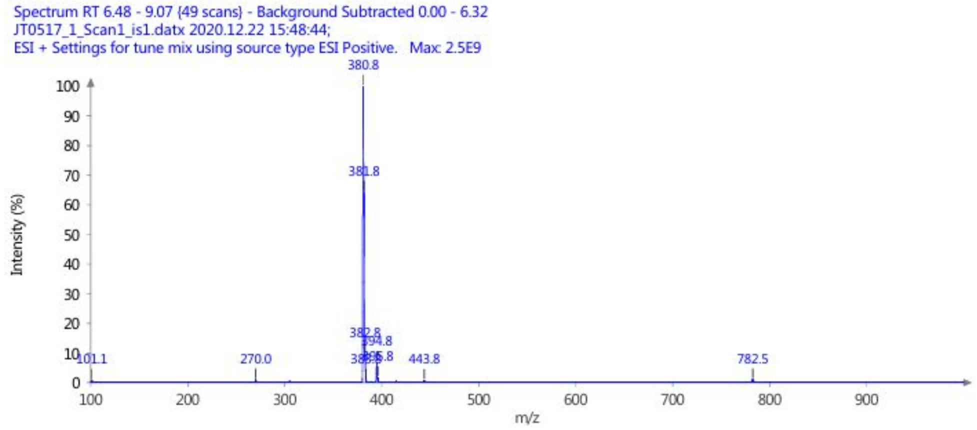

Spectrum RT 6.48 - 9.07 (49 scans) - Background Subtracted 0.00 - 6.32
JT0517_1_Scan1_is1.datx 2020.12.22 15:48:44;
ESI + Settings for tune mix using source type ESI Positive. Max: 2.5E9
380.8
381.8
382.8
394.8
443.8
101.1
270.0
782.5
Intensity (%)
m/z

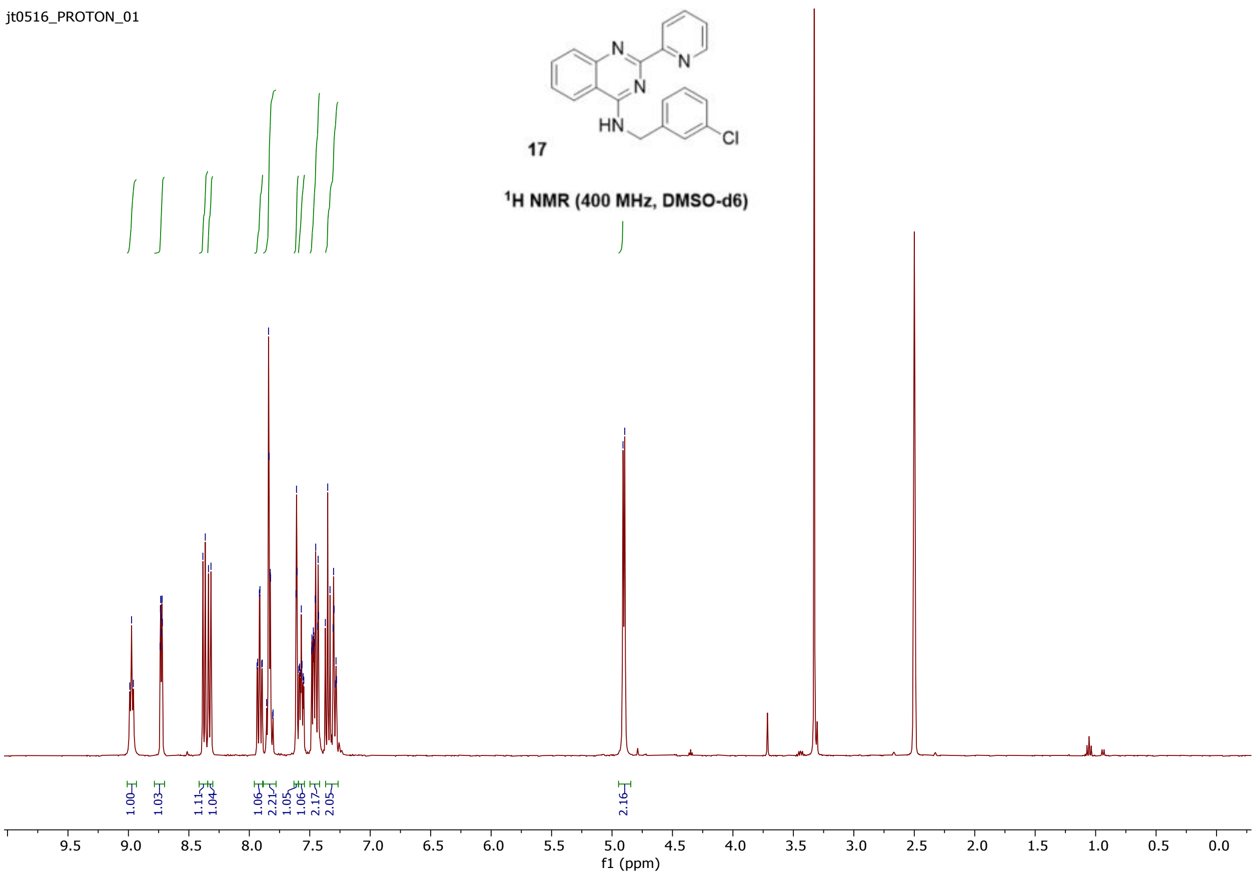

17 HPLC

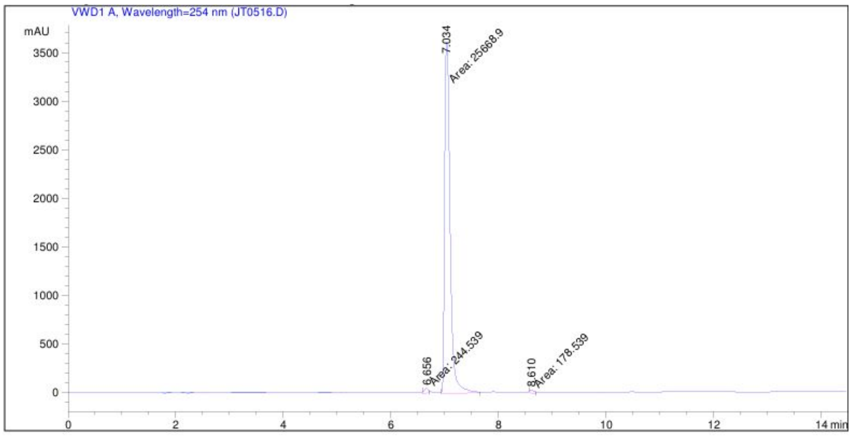


```
=====================================================================
                          Area Percent Report
=====================================================================

Sorted By             :      Signal
Multiplier            :      1.0000
Dilution              :      1.0000
Use Multiplier & Dilution Factor with ISTDs


Signal 1: VWD1 A, Wavelength=254 nm

Peak RetTime Type  Width      Area       Height      Area
  #   [min]        [min]  mAU    *s    [mAU    ]      %
----|-------|----|-------|----------|----------|--------|
   1   6.656 MM    0.0826  244.53935   49.33880   0.9372
   2   7.034 MM    0.1182 2.56689e4  3618.69922  98.3785
   3   8.610 MM    0.0947  178.53938   31.43825   0.6843

Totals :                 2.60920e4  3699.47627


=====================================================================
                          *** End of Report ***
```

17 LCMS

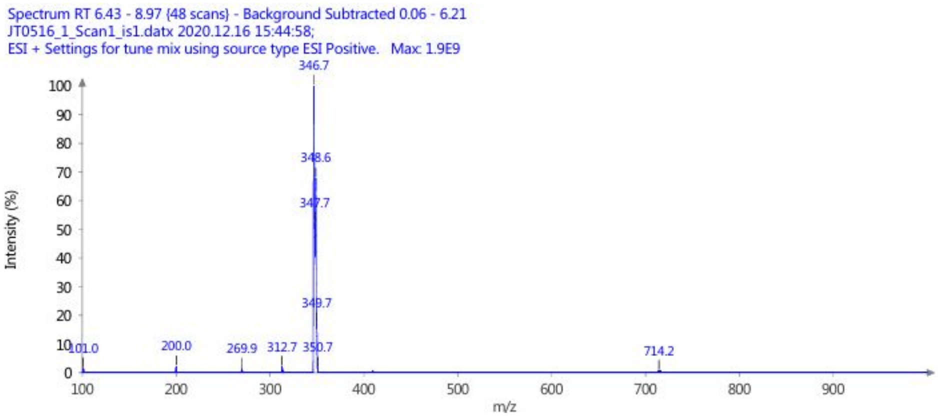

Spectrum RT 6.43 - 8.97 (48 scans) - Background Subtracted 0.06 - 6.21
JT0516_1_Scan1_is1.datx 2020.12.16 15:44:58;
ESI + Settings for tune mix using source type ESI Positive. Max: 1.9E9
346.7
348.6
347.7
349.7
101.0
200.0
269.9
312.7
350.7
714.2
Intensity (%)
m/z

References

1. Adapted from S.P. Flanagan et al., Tetrahedron, 2005, vol. 61(41), pp. 9808-9821.
2. WO2006-EP60643
